# Supplementary material for: Cytotoxic and Antibacterial Prenylated Acylphloroglucinols from Hypericum olympicum L
Source: Plants (Basel). 2023 Mar 29;12(7):1500. doi: 10.3390/plants12071500 (PMC10097024; doi:10.3390/plants12071500)
Supplement: Supplementary file 1 [file plants-12-01500-s001.zip › plants-2313642-supplementary.pdf]

Content

Figure S1. HRESIMS spectrum of compound 1. 2
Figure S2. MS/MS spectrum of the deprotonated molecule $[M-H]^-$ of compound 1. 2
Figure S3. ^1H-NMR spectrum of compound 1. 3
Figure S4. ^{13}C-NMR spectrum of compound 1. 3
Figure S5. ^1H-^1H COSY experiment of compound 1. 4
Figure S6. HSQC experiment of compound 1. 4
Figure S7. HMBC experiment of compound 1. 5
Figure S8. NOESY experiment of compound 1. 5
Figure S9. HRESIMS spectrum of compound 4. 6
Figure S10. MS/MS spectrum of the protonated molecule $[M+H]^+$ of compound 4. 6
Figure S11. ^1H-NMR spectrum of compound 4. 7
Figure S12. ^{13}C-NMR spectrum of compound 4. 7
Figure S13. ^1H-^1H COSY experiment of compound 4. 8
Figure S14. HSQC experiment of compound 4. 8
Figure S15. HMBC experiment of compound 4. 9
Figure S16. NOESY experiment of compound 4. 9
Figure S17. HRESIMS spectrum of compound 4a. 10
Figure S18. MS/MS spectrum of the protonated molecule $[M+H]^+$ of compound 4a. 10
Figure S19. ^1H-NMR spectrum of compound 4a. 11
Figure S20. ^{13}C-NMR spectrum of compound 4a. 11
Figure S21. ^1H-^1H COSY experiment of compound 4a. 12
Figure S22. HSQC experiment of compound 4a. 12
Figure S23. HMBC experiment of compound 4a. 13
Figure S24. NOESY experiment of compound 4a. 13
Figure S25. HRESIMS spectrum of compound 4b. 14
Figure S26. MS/MS spectrum of the protonated molecule $[M+H]^+$ of compound 4b. 14
Figure S27. ^1H-NMR spectrum of compound 4b. 15
Figure S28. ^{13}C-NMR spectrum of compound 4b. 15
Figure S29. ^1H-^1H COSY experiment of compound 4a. 16
Figure S30. HSQC experiment of compound 4b. 16
Figure S31. HMBC experiment of compound 4b. 17
Figure S32. NOESY experiment of compound 4b. 17
Figure S33. HRESIMS spectrum of compound 5. 18
Figure S34. MS/MS spectrum of the protonated molecule $[M+H]^+$ of compound 5. 18
Figure S35. ^1H-NMR spectrum of compound 5. 19
Figure S36. ^{13}C-NMR spectrum of 5. 19
Figure S37. ^1H-^1H COSY experiment of compound 5. 20
Figure S38. HSQC experiment of 5. 20
Figure S39. HMBC experiment of compound 5. 21
Figure S40. NOESY experiment of compound 5. 21
Table S1. Absorbance at 550 nm of the MRSA biofilm died with Hucker crystal violet after exposure to the tested compounds. 22
Table S2. One-way ANOVA of the metabolic activity of the tested strains after exposure to hyperpolyphyllirin/hyperibine J (3), olympiforin A (4) and olympiforin B (5). Comparison between the treated groups and untreated control. 23

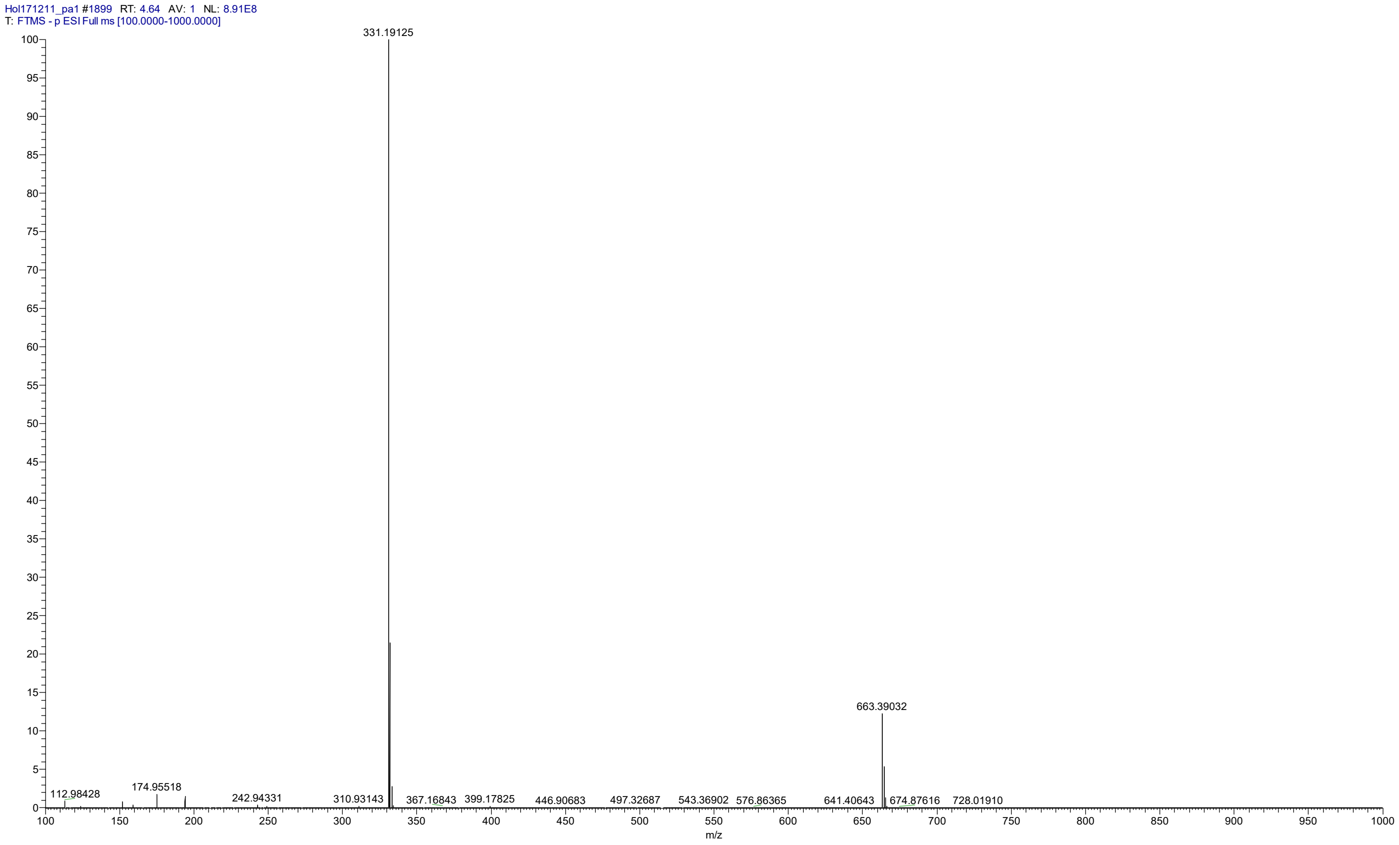

Figure S1. HRESIMS spectrum of compound **1**.

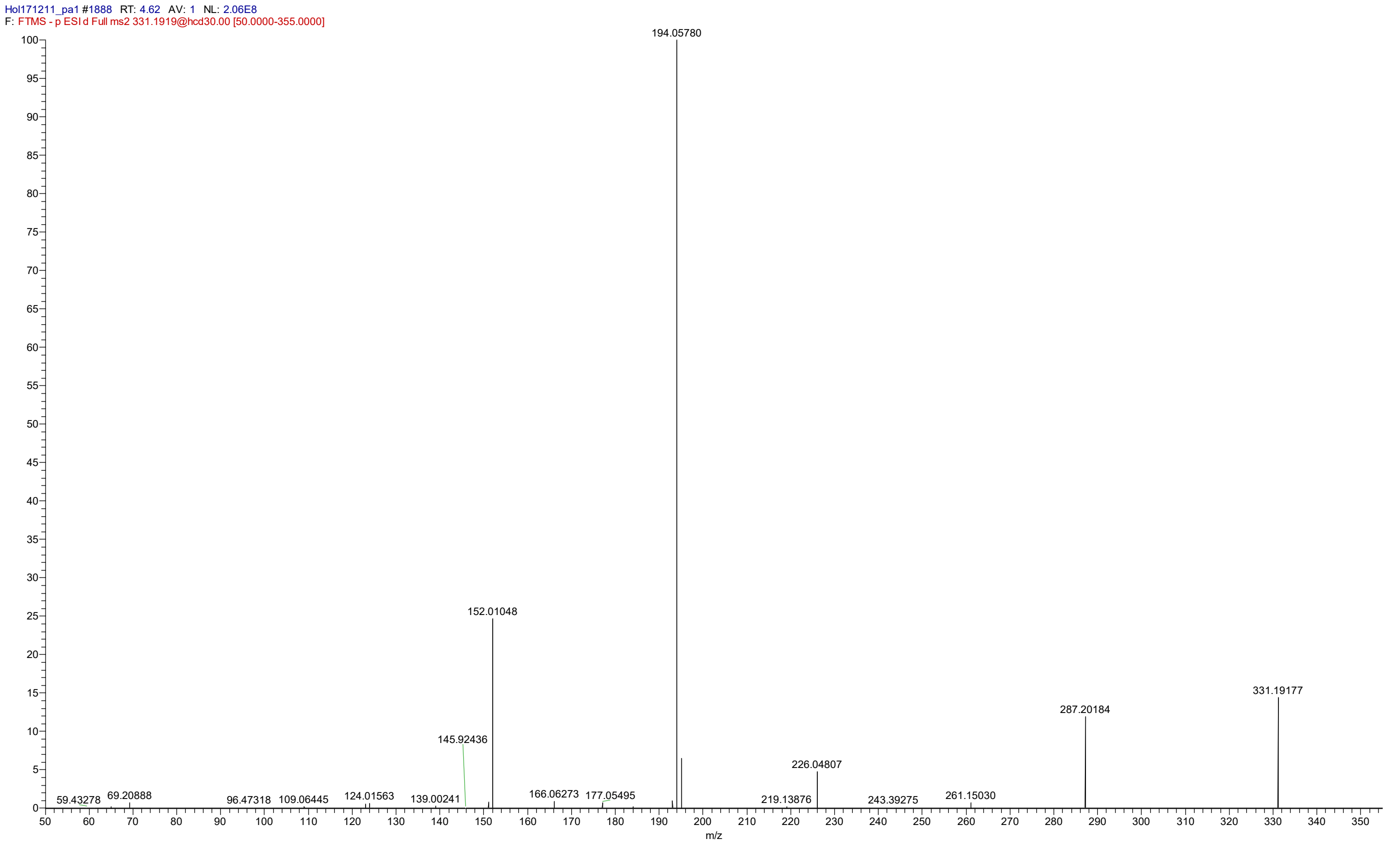

Figure S2. MS/MS spectrum of the deprotonated molecule $[M-H]^-$ of compound **1**.

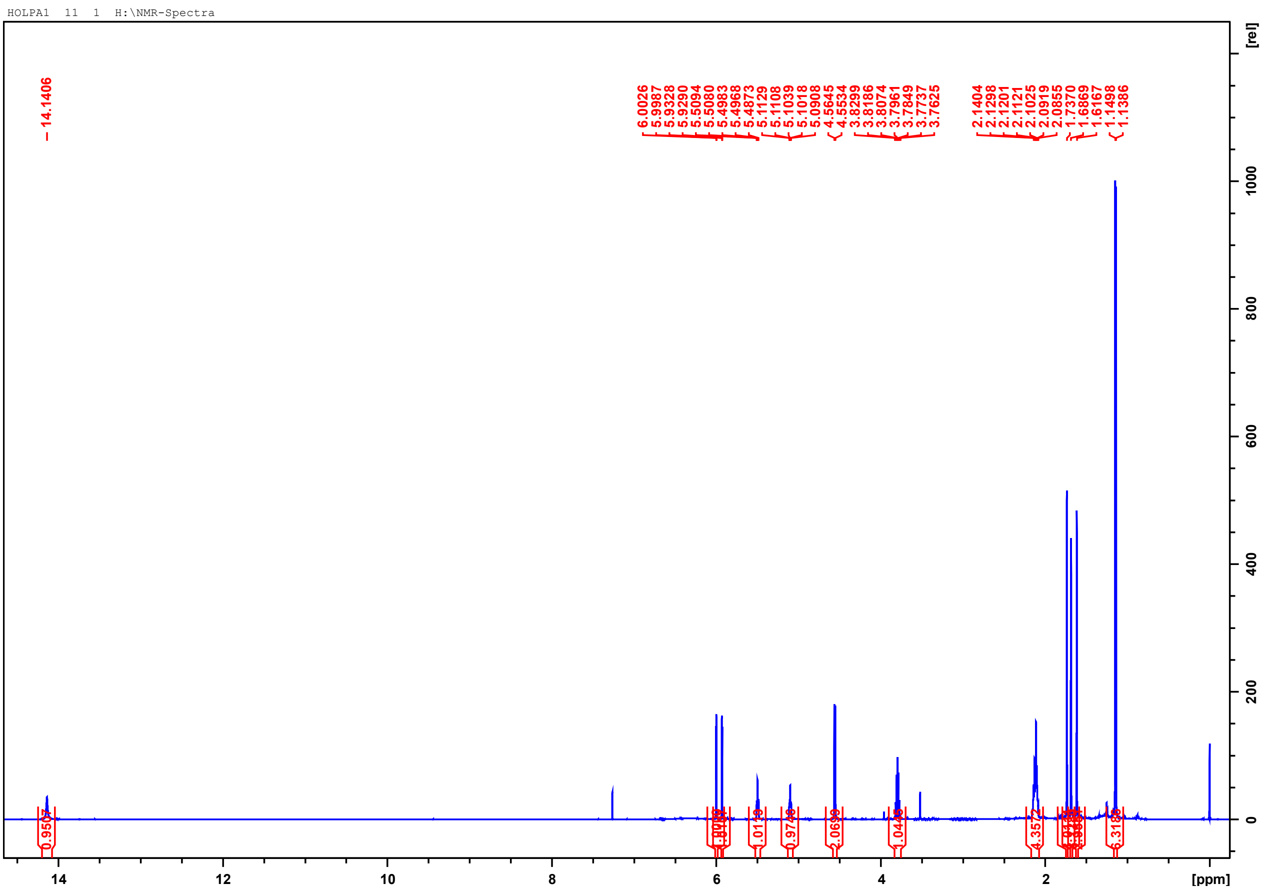

Figure S3. ^{1}H-NMR spectrum of compound **1**.

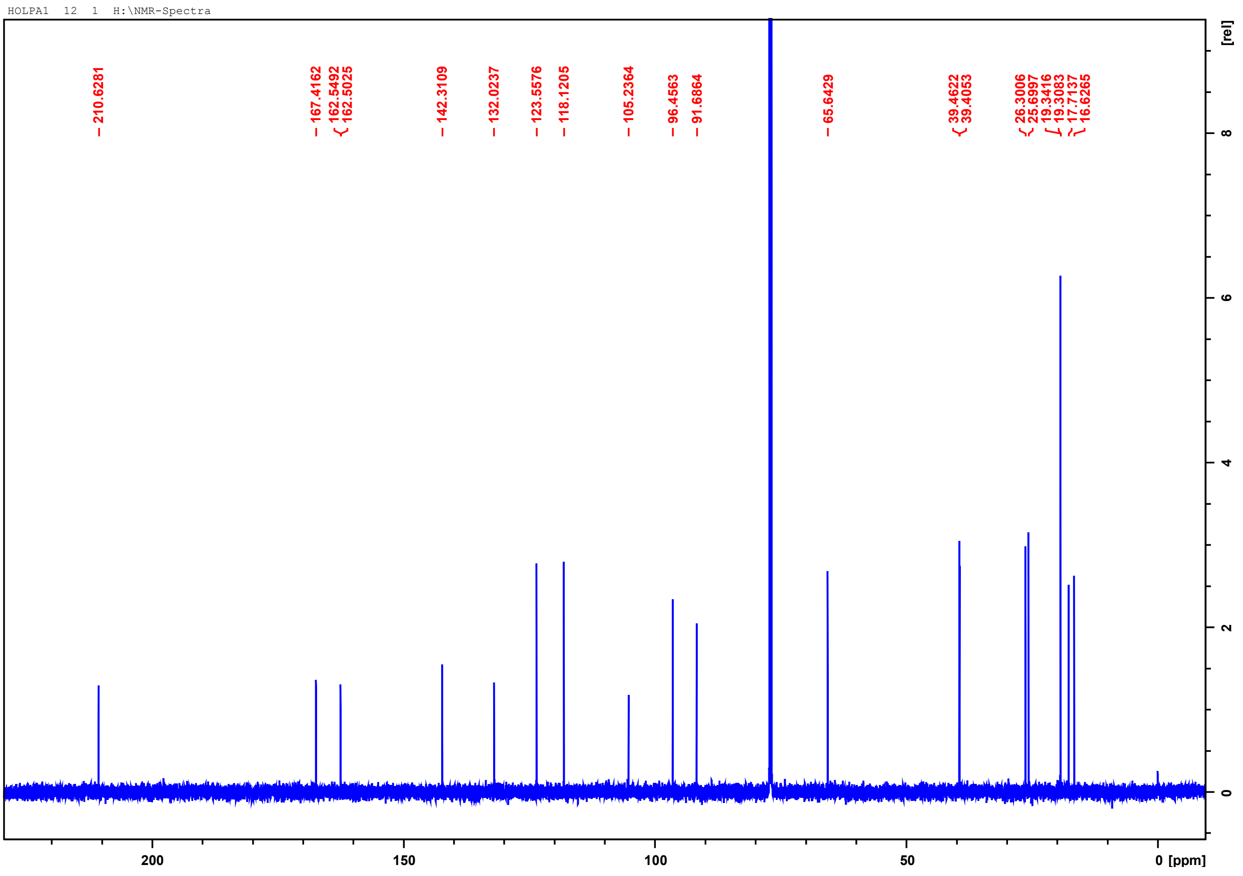

Figure S4. ^{13}C-NMR spectrum of compound **1**.

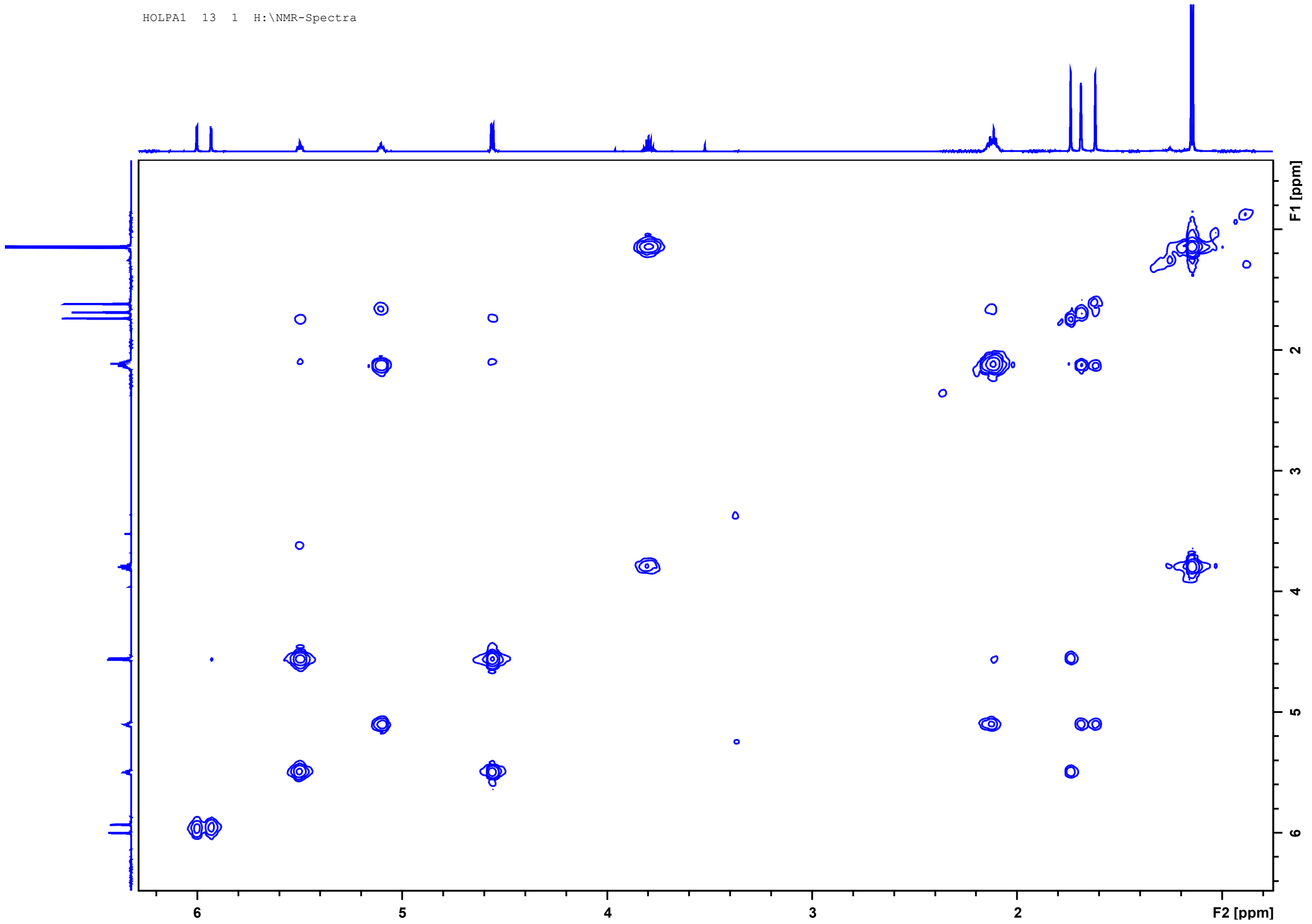

Figure S5. ^{1}H-^{1}H COSY experiment of compound **1**.

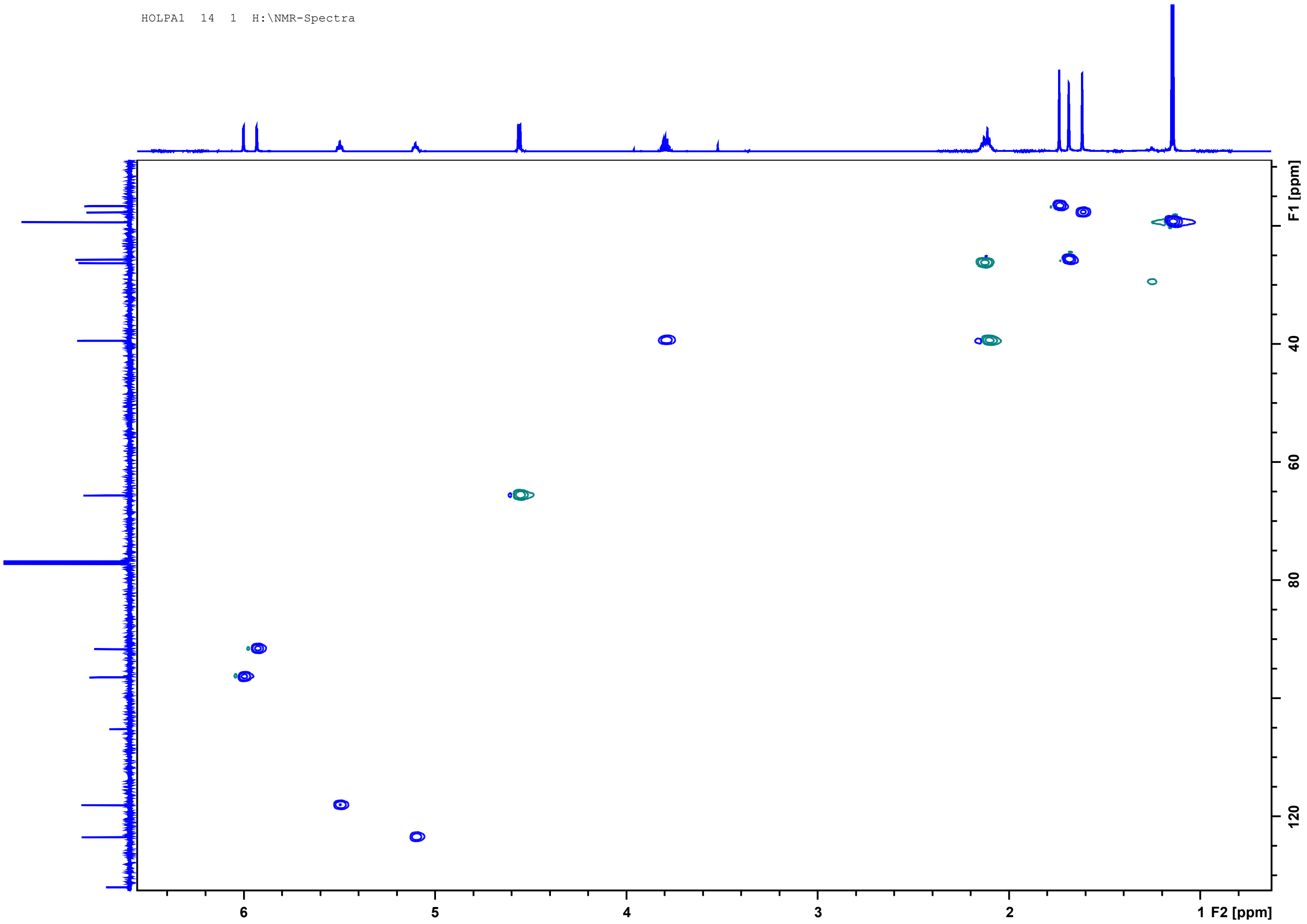

Figure S6. HSQC experiment of compound **1**.

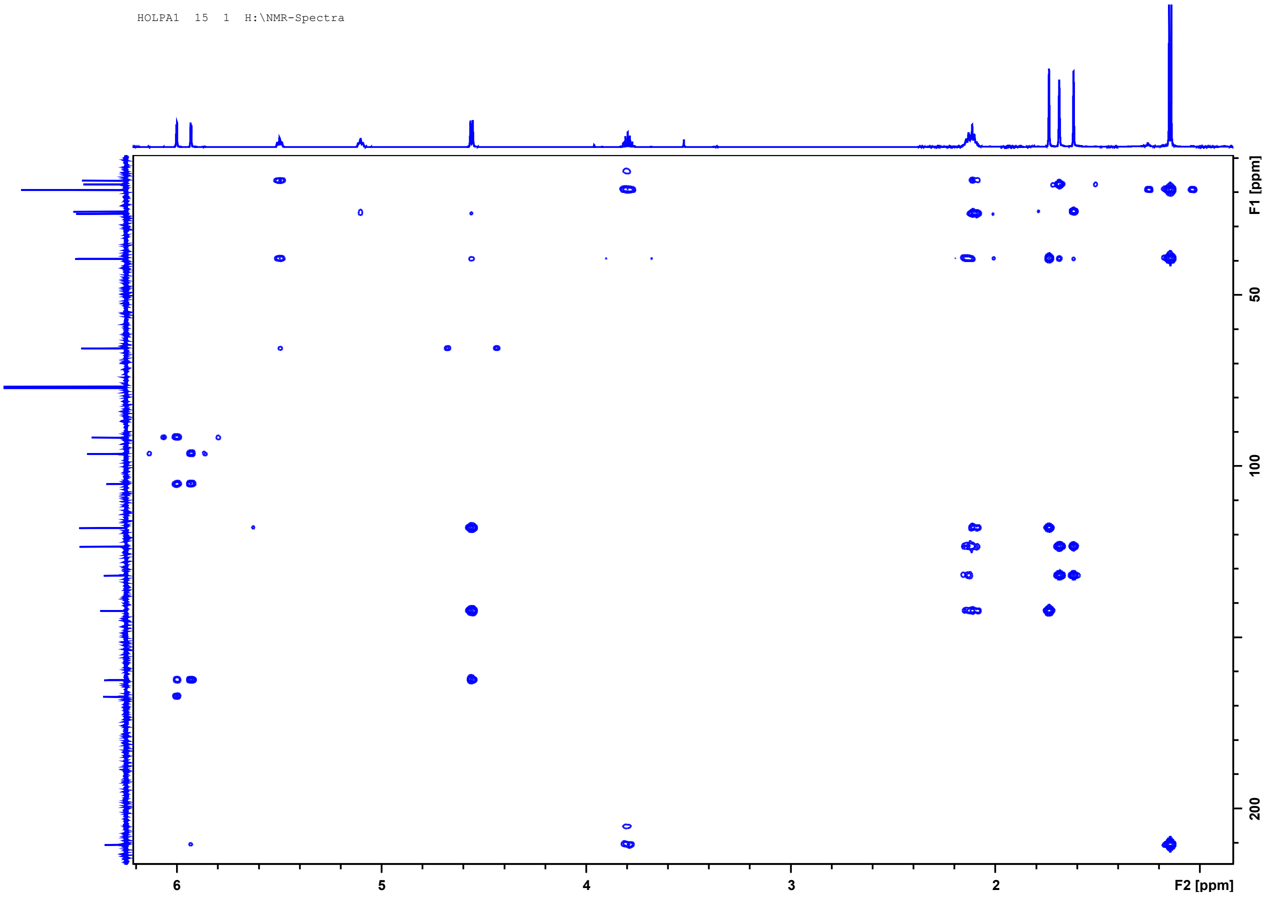

Figure S7. HMBC experiment of compound **1**.

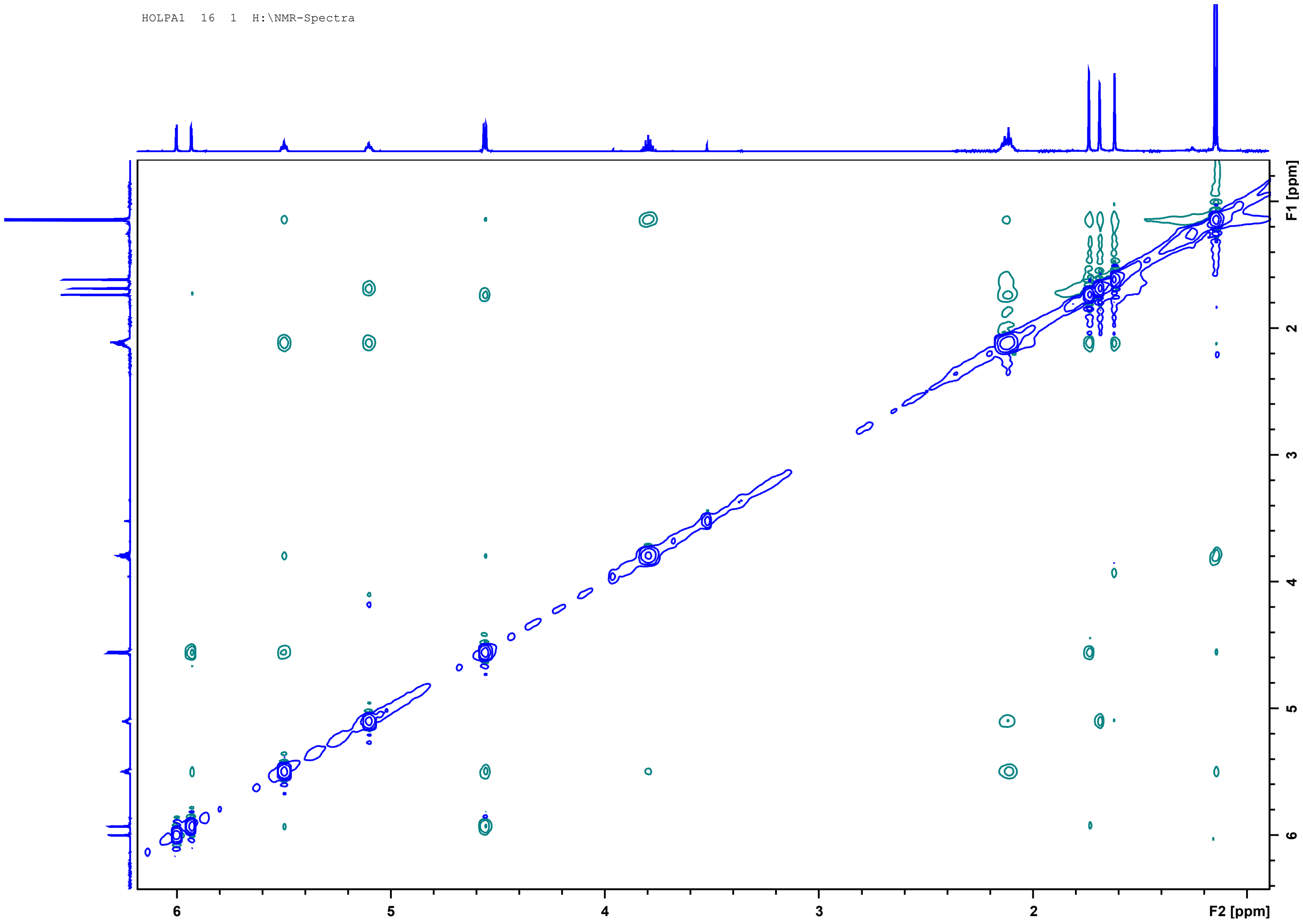

Figure S8. NOESY experiment of compound **1**.

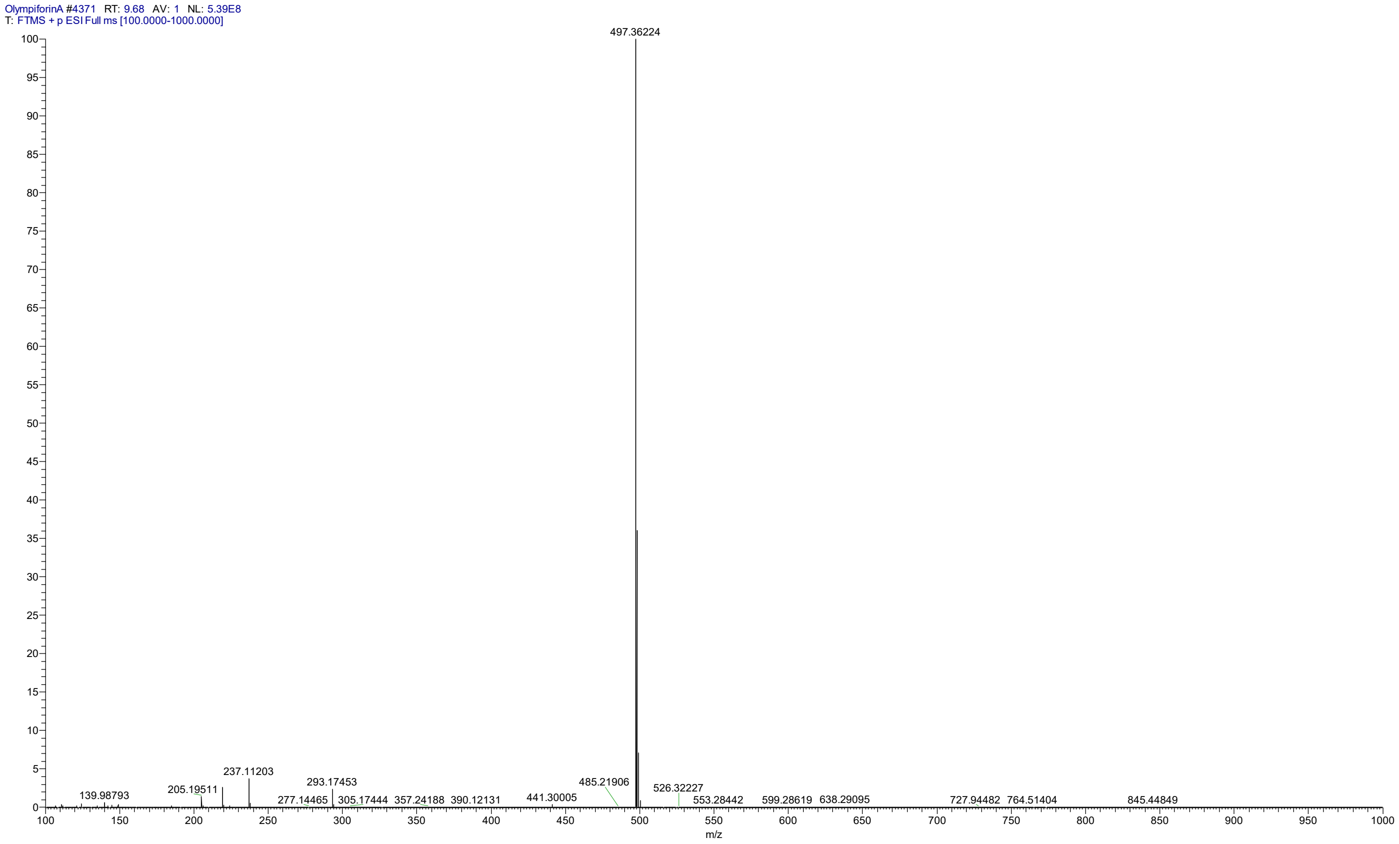

Figure S9. HRESIMS spectrum of compound **4**.

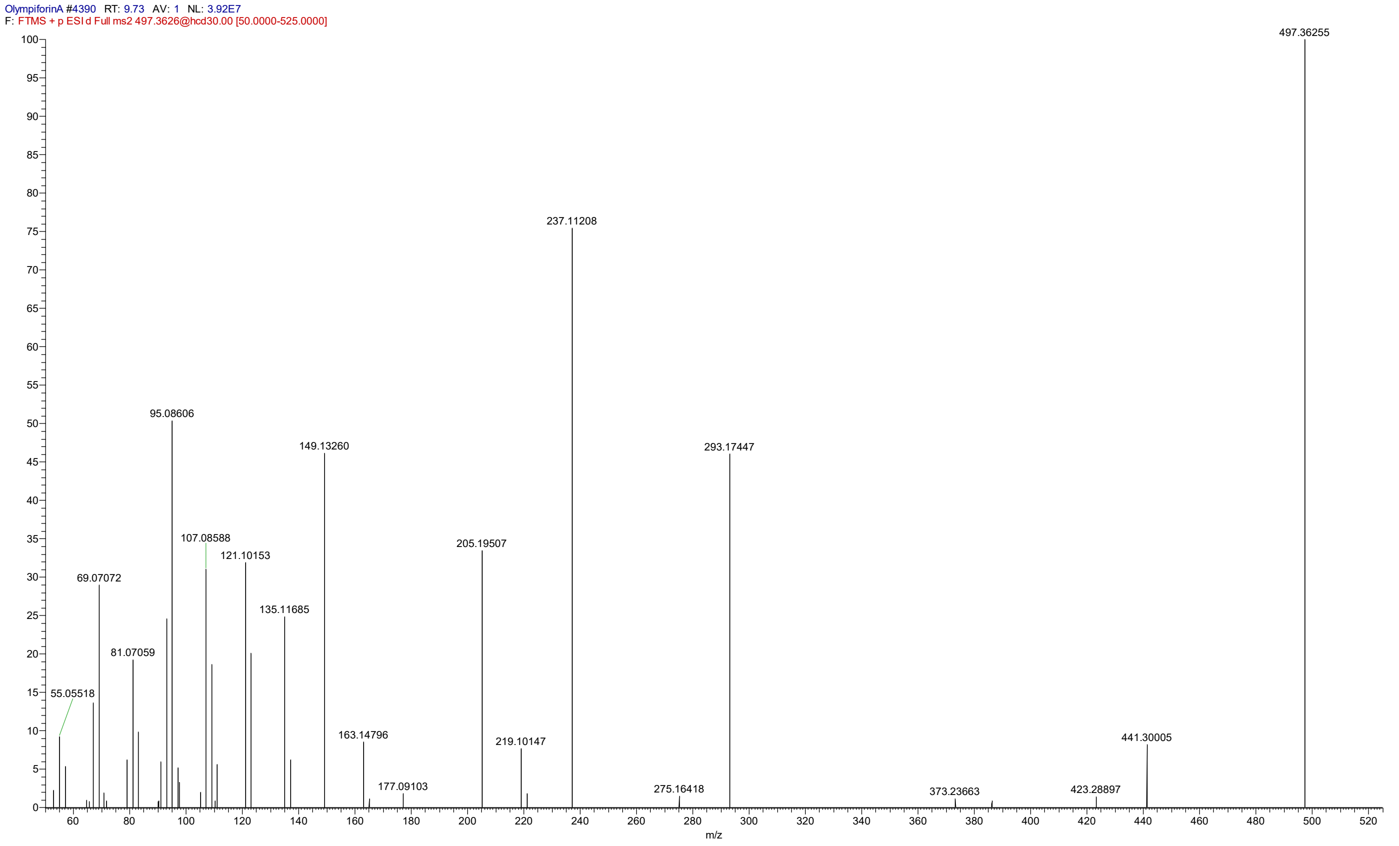

Figure S10. MS/MS spectrum of the protonated molecule $[M+H]^+$ of compound **4**.

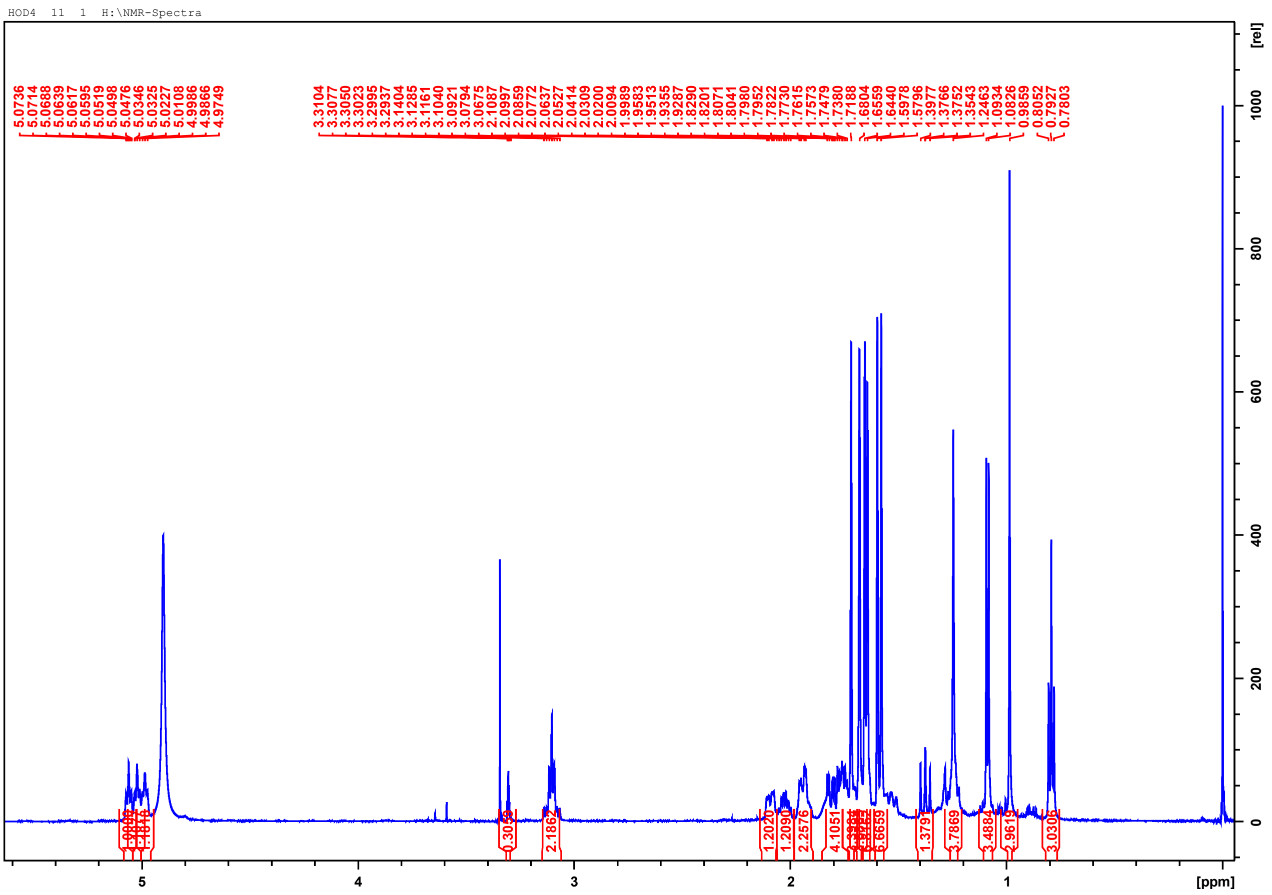

Figure S11. ^{1}H-NMR spectrum of compound **4**.

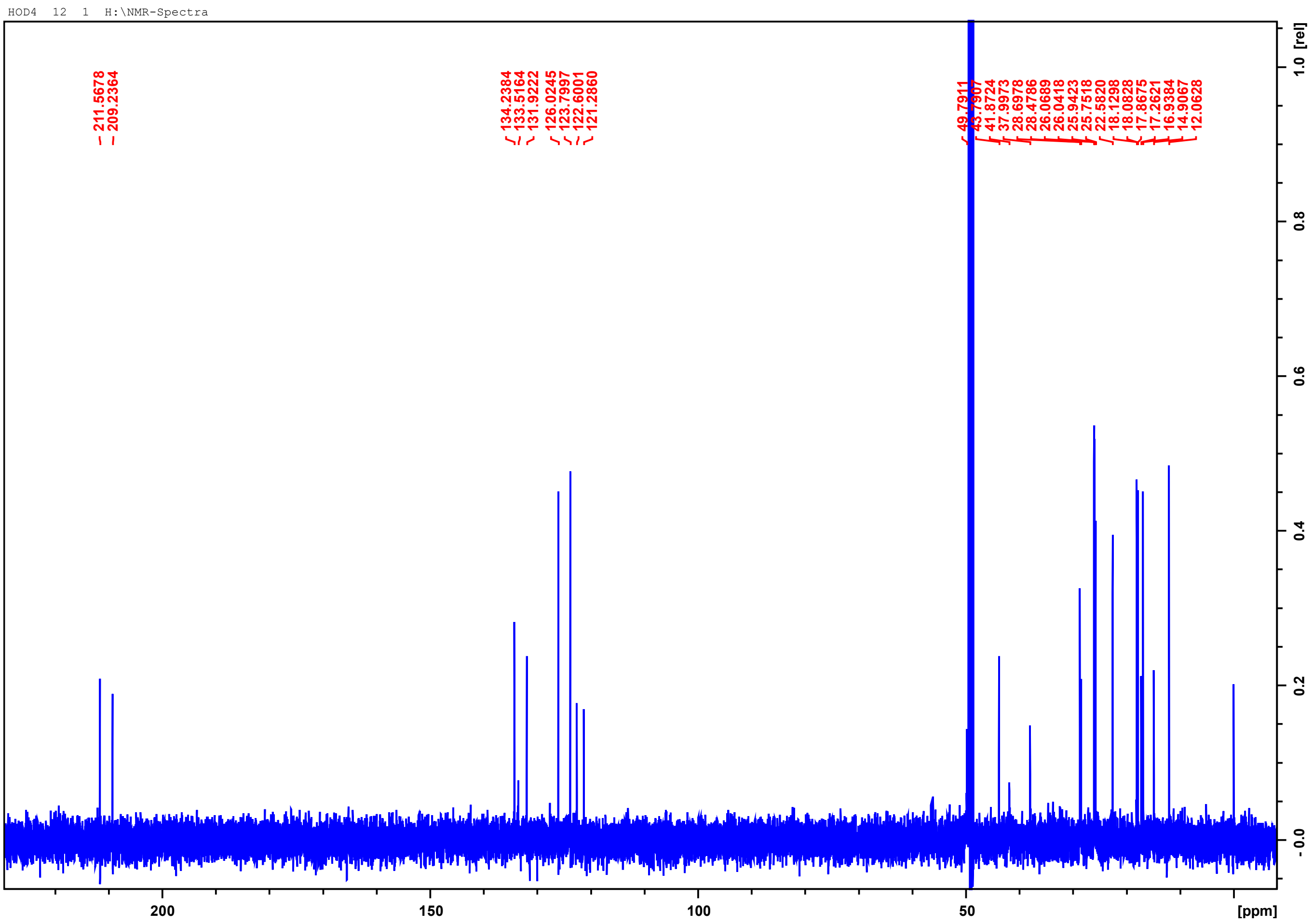

Figure S12. ^{13}C-NMR spectrum of compound **4**.

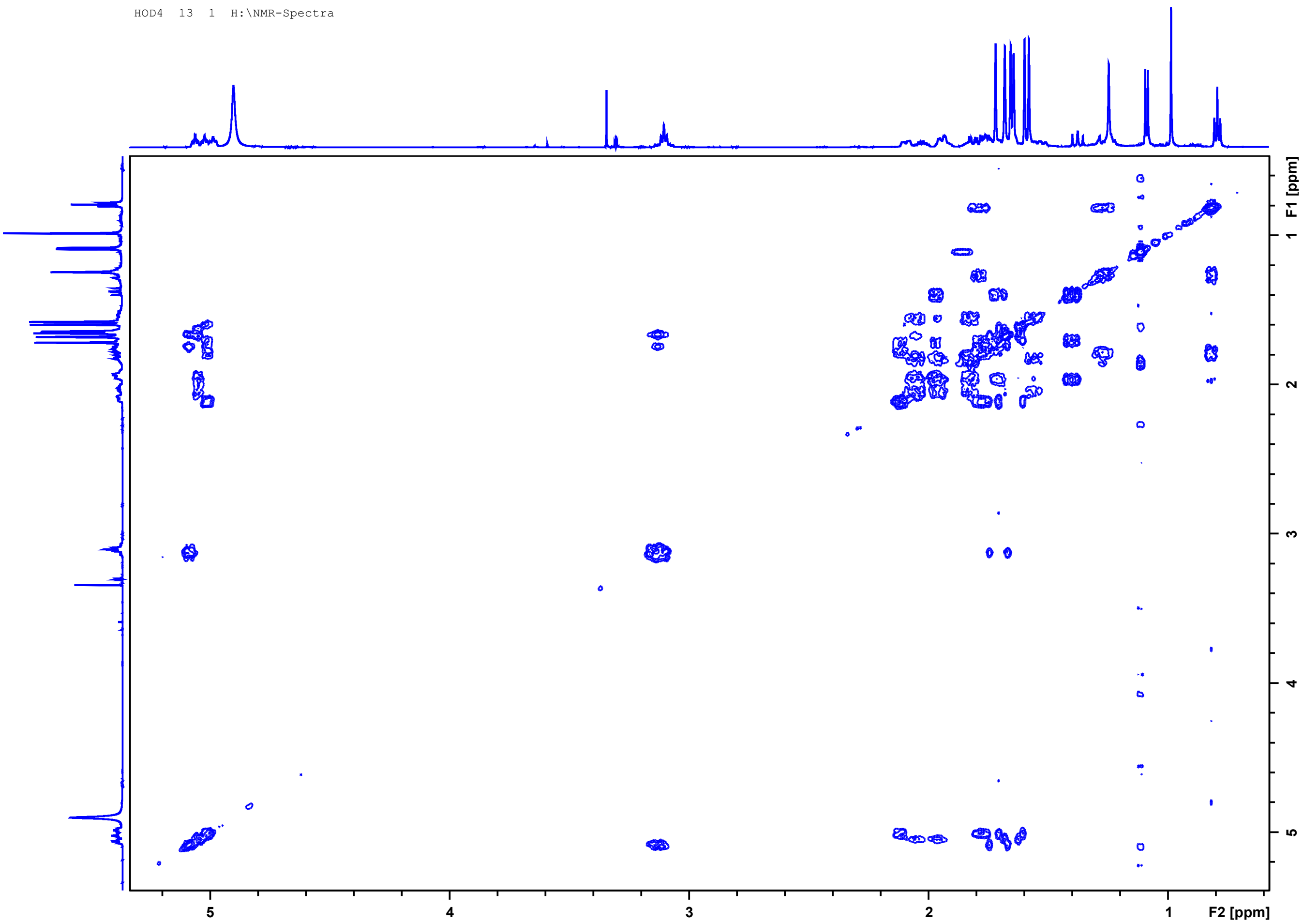

Figure S13. ^1H-^1H COSY experiment of compound **4**.

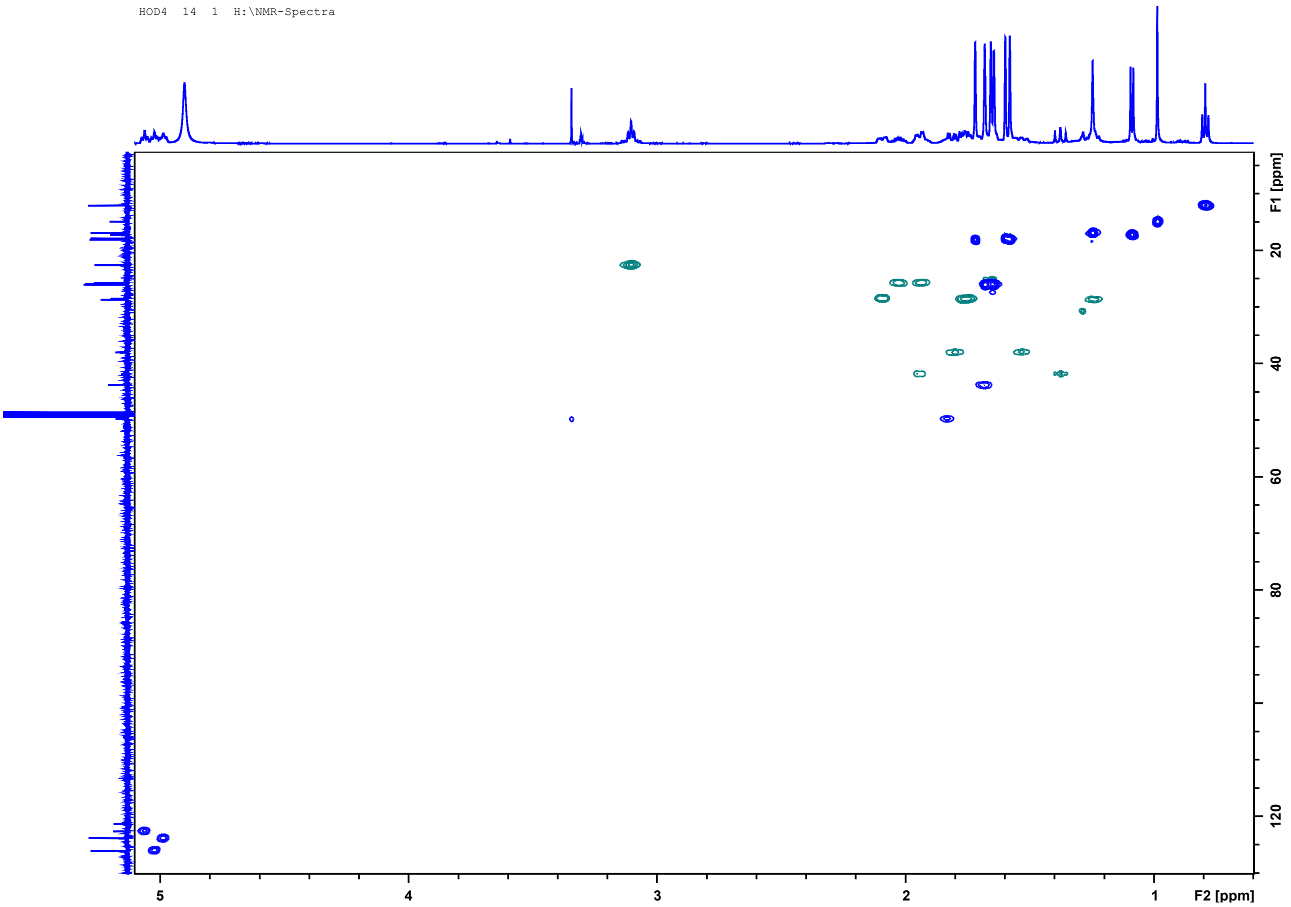

Figure S14. HSQC experiment of compound **4**.

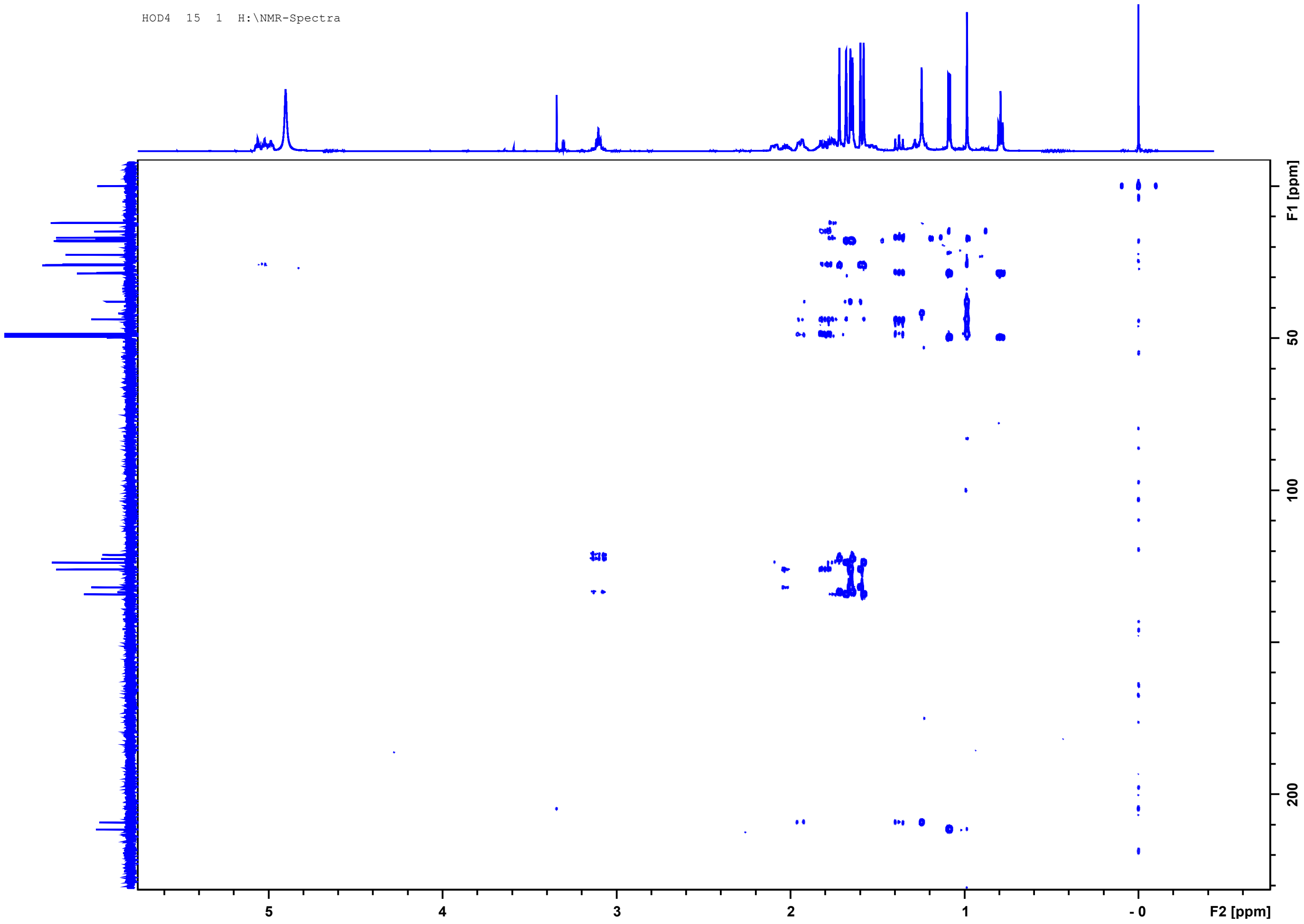

Figure S15. HMBC experiment of compound **4**.

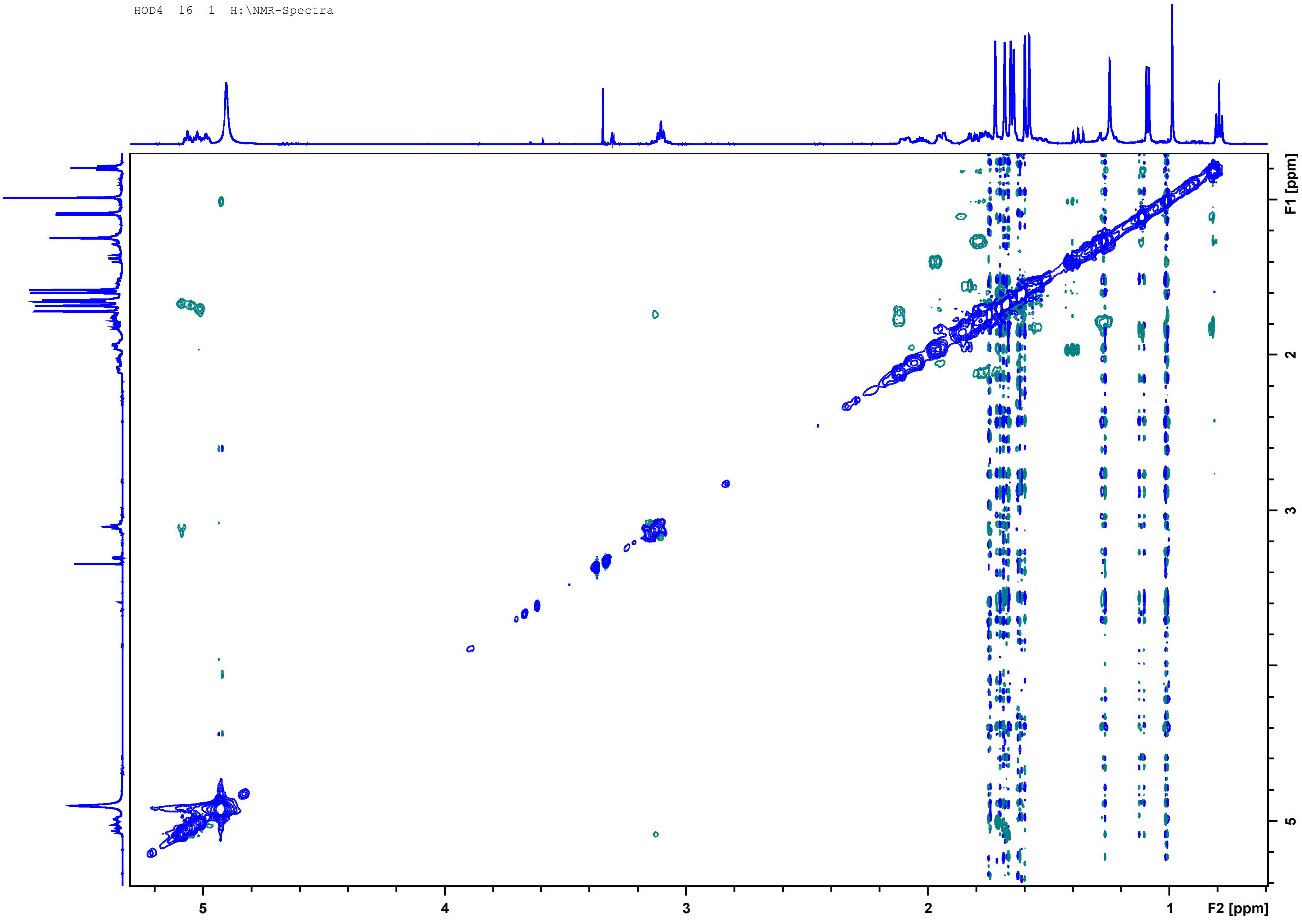

Figure S16. NOESY experiment of compound **4**.

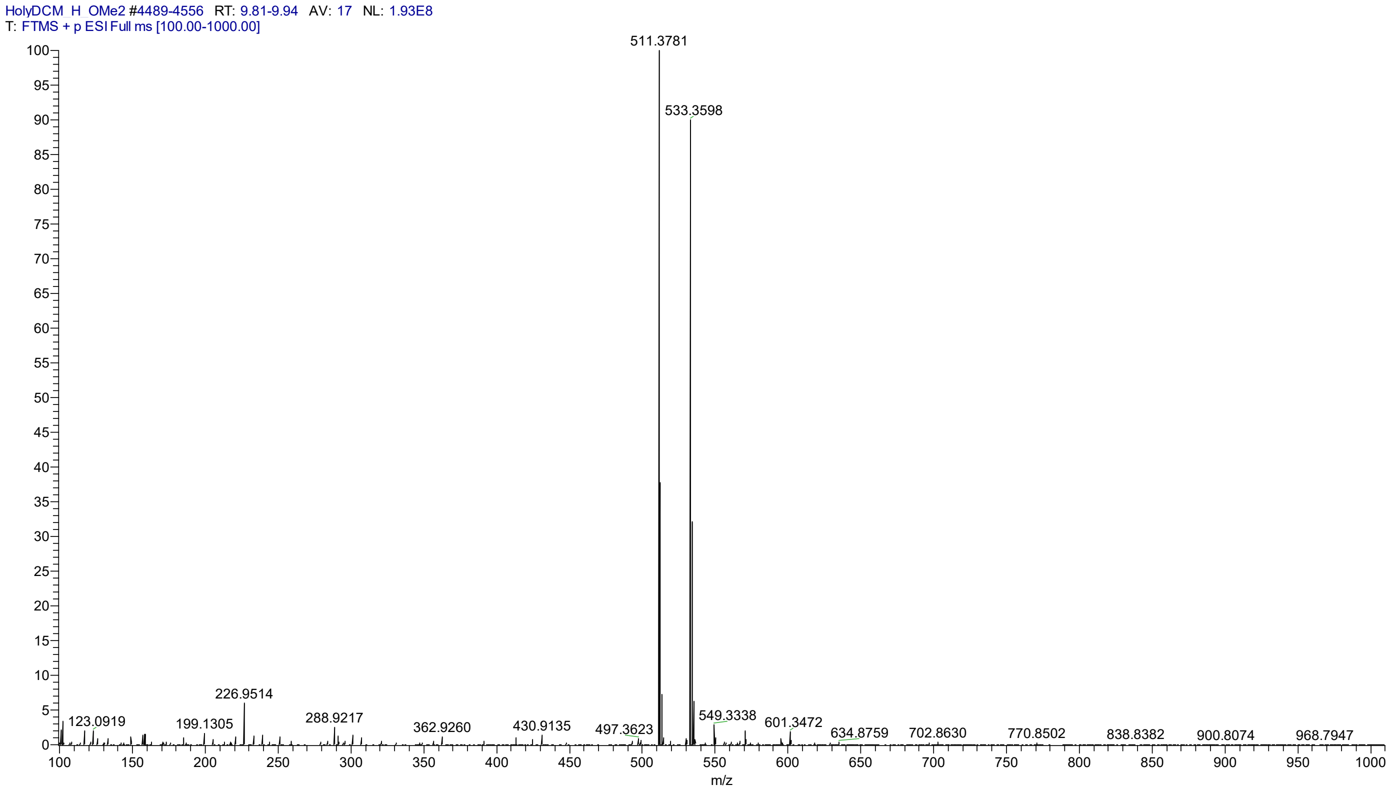

Figure S17. HRESIMS spectrum of compound **4a**.

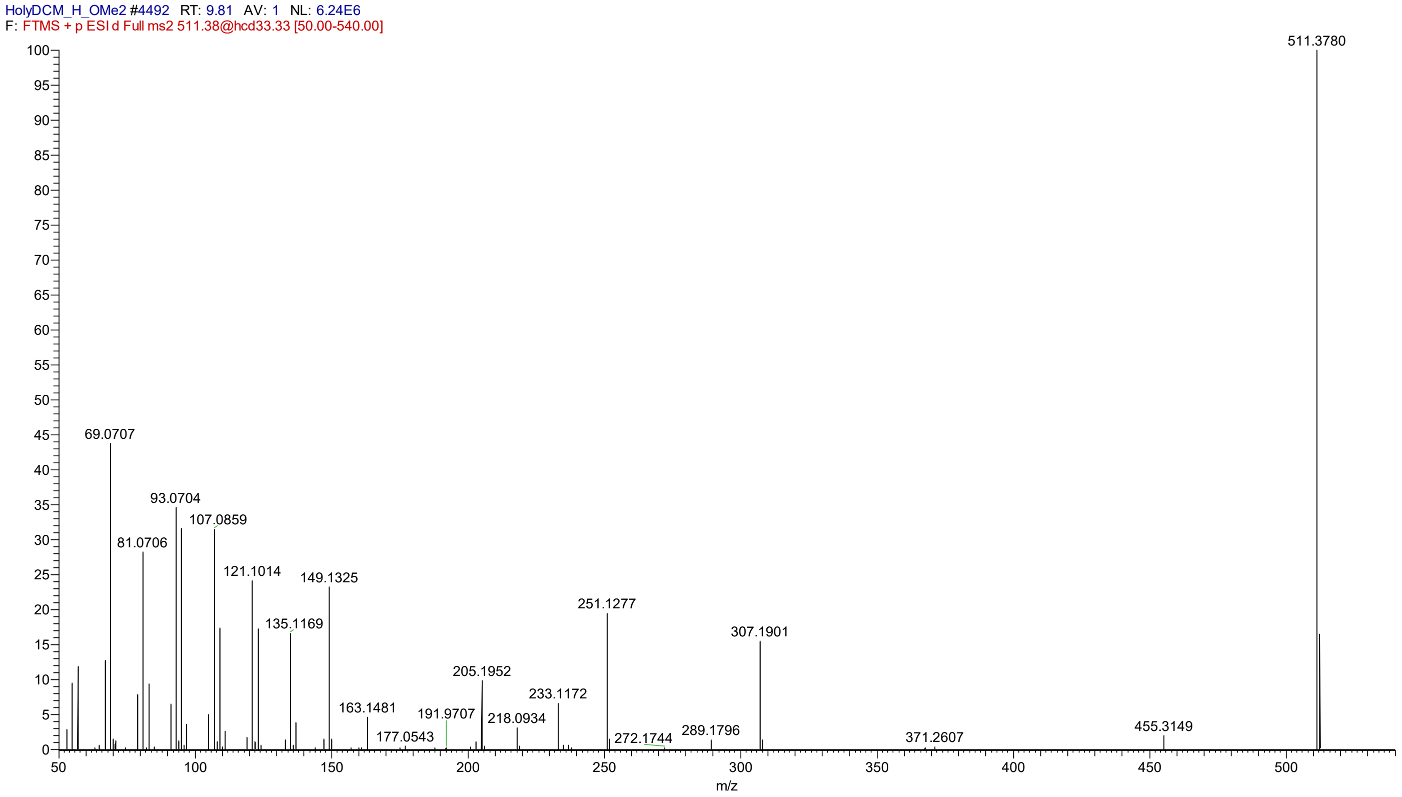

Figure S18. MS/MS spectrum of the protonated molecule $[M+H]^+$ of compound **4a**.

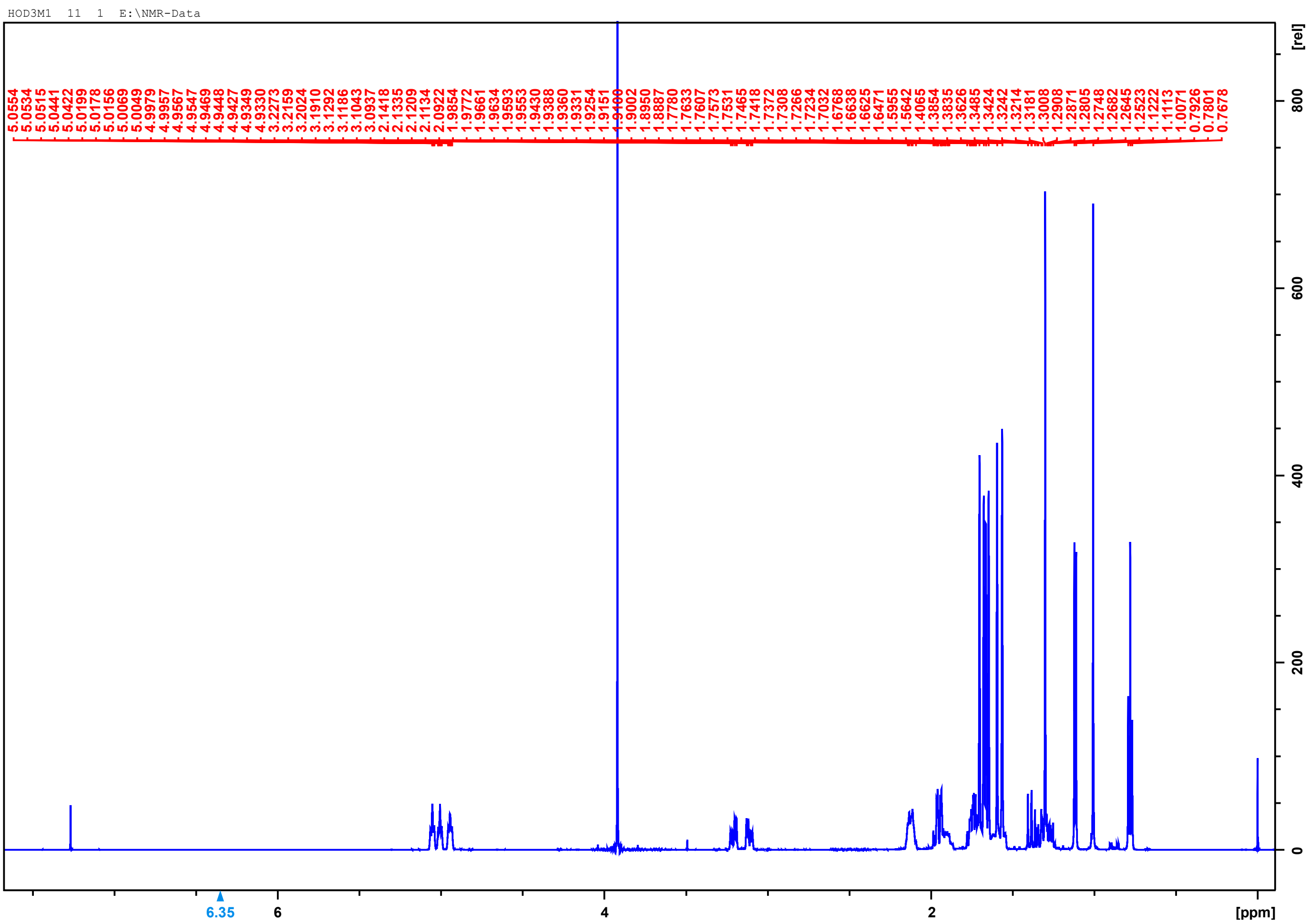

Figure S19. ^{1}H-NMR spectrum of compound **4a**.

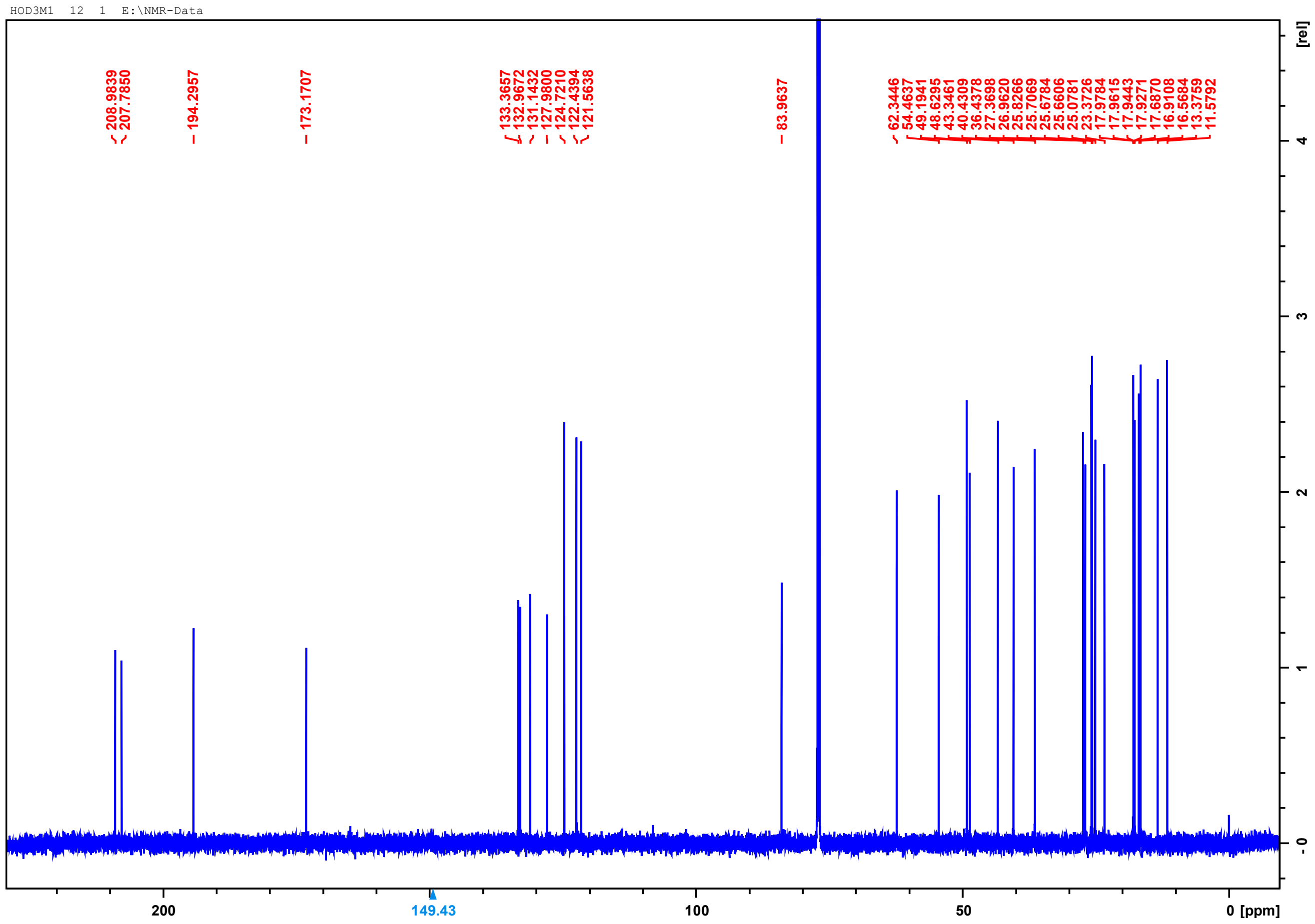

Figure S20. ^{13}C-NMR spectrum of compound **4a**.

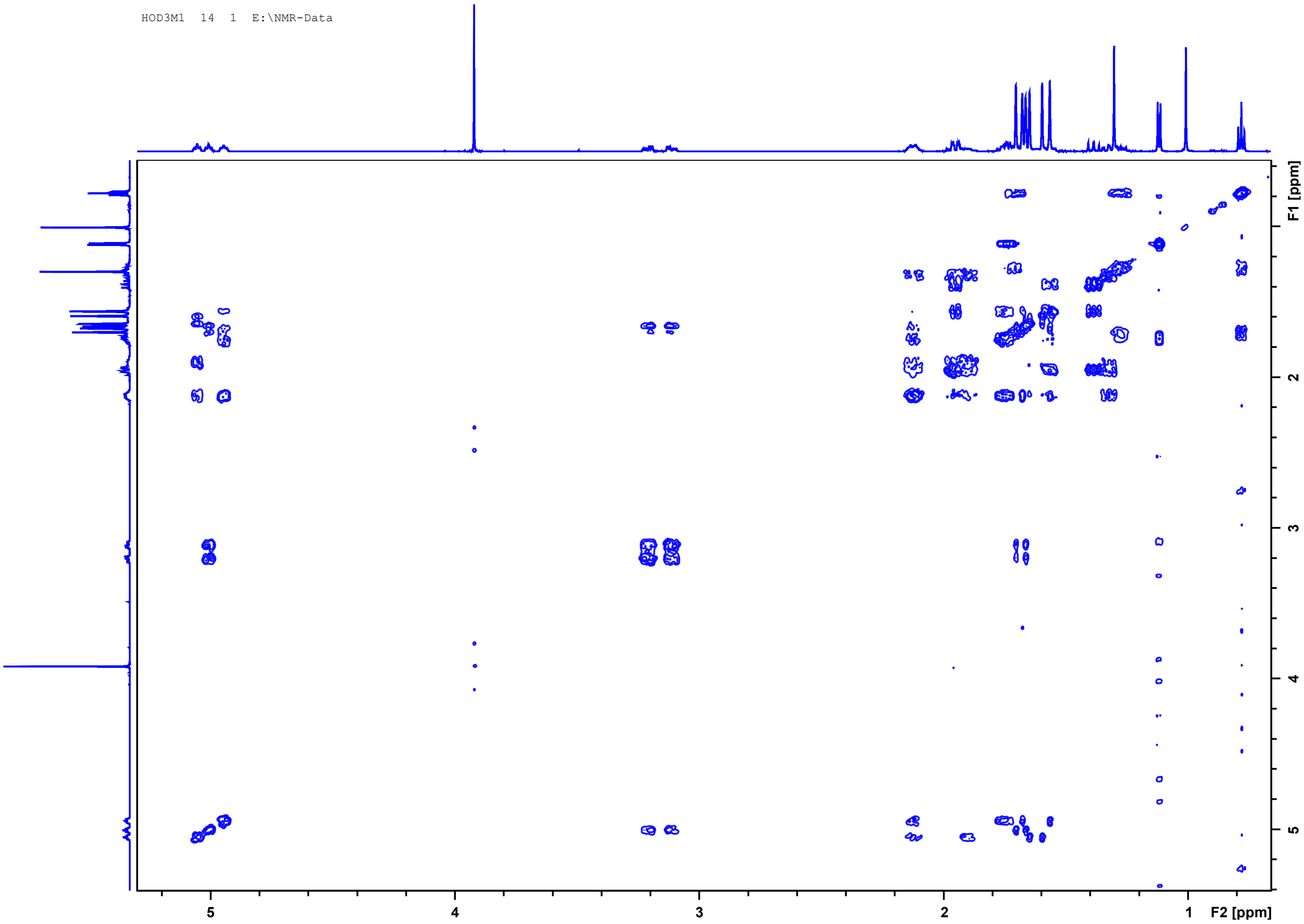

Figure S21. ^{1}H-^{1}H COSY experiment of compound **4a**.

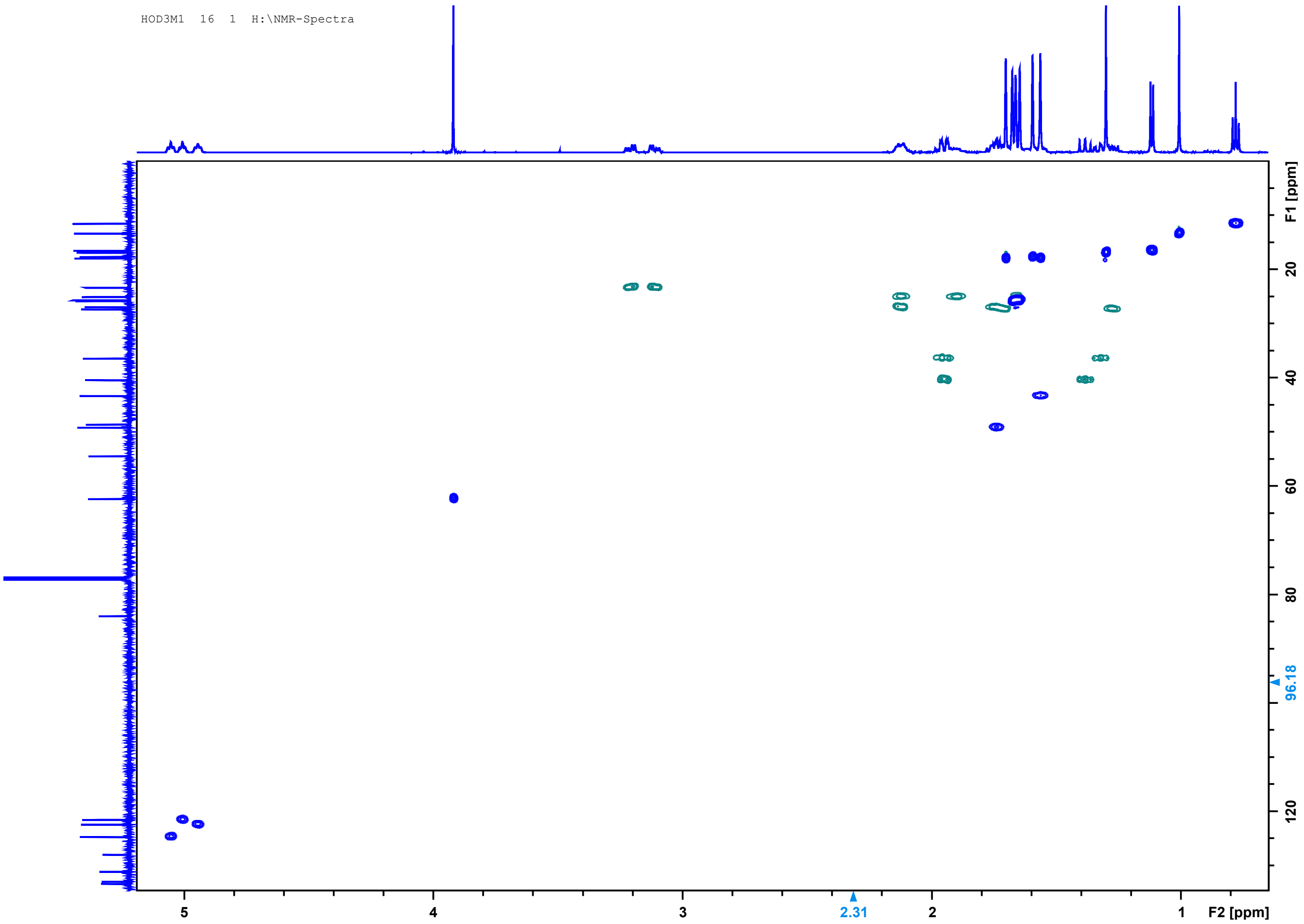

Figure S22. HSQC experiment of compound **4a**.

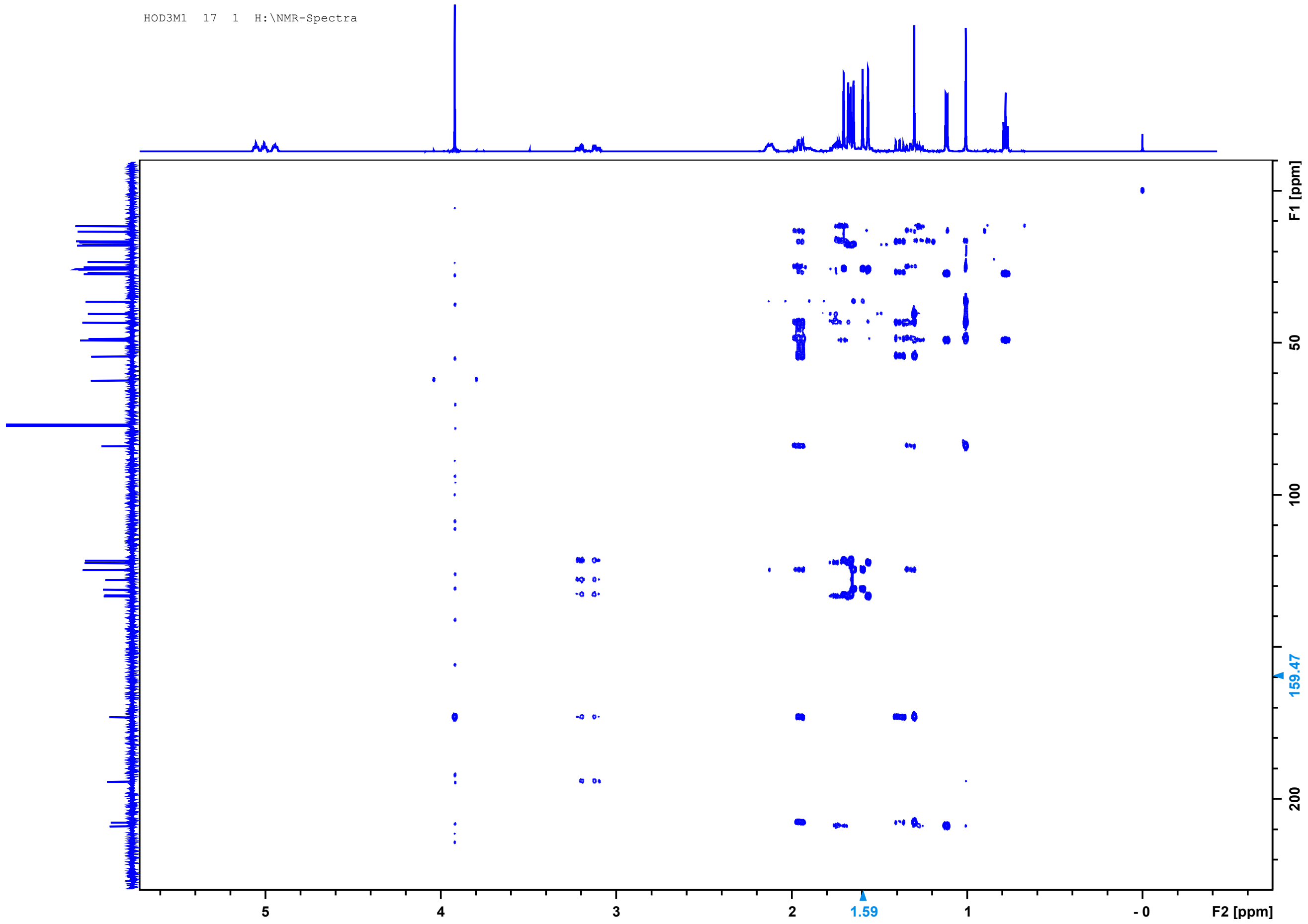

Figure S23. HMBC experiment of compound **4a**.

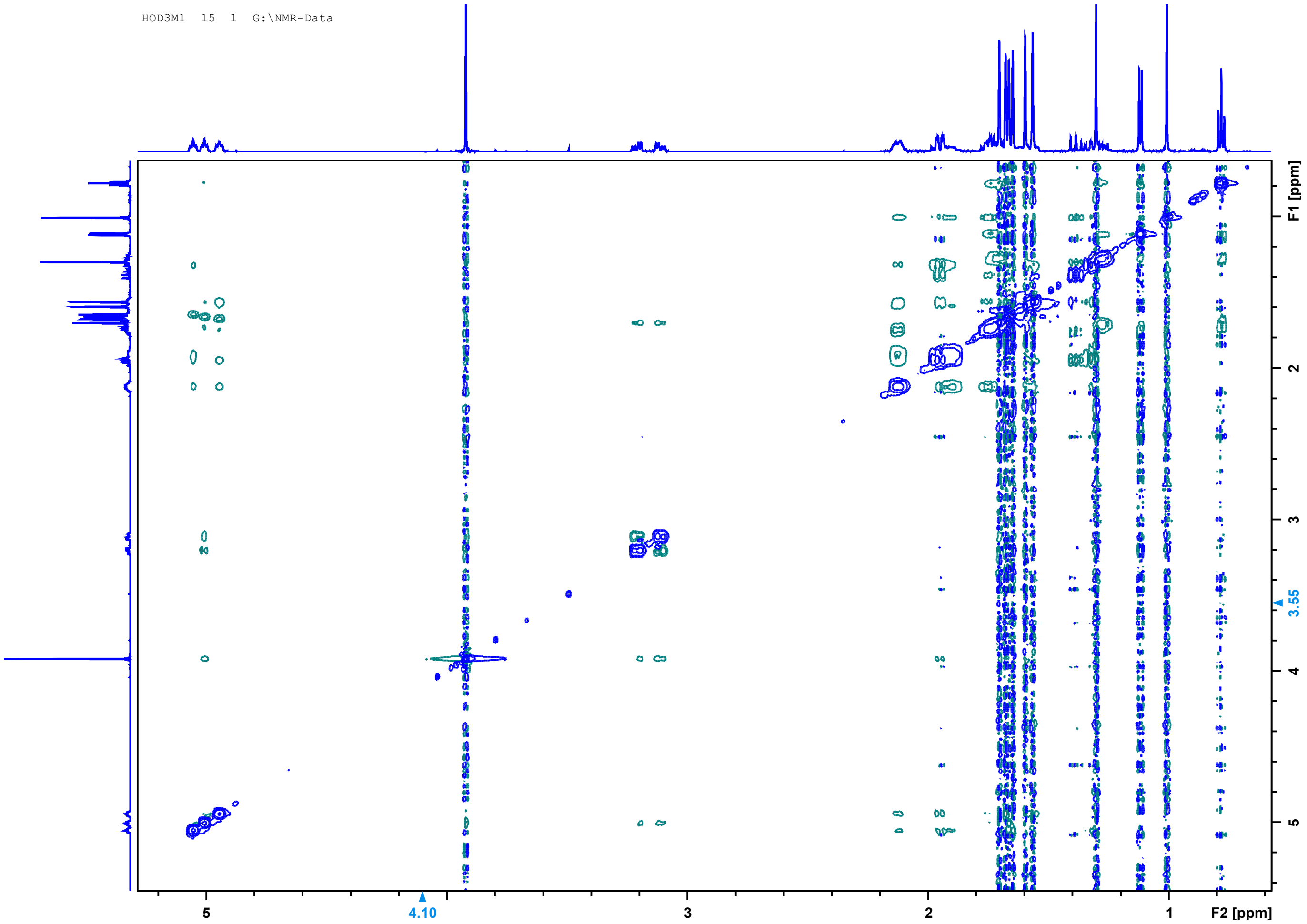

Figure S24. NOESY experiment of compound **4a**.

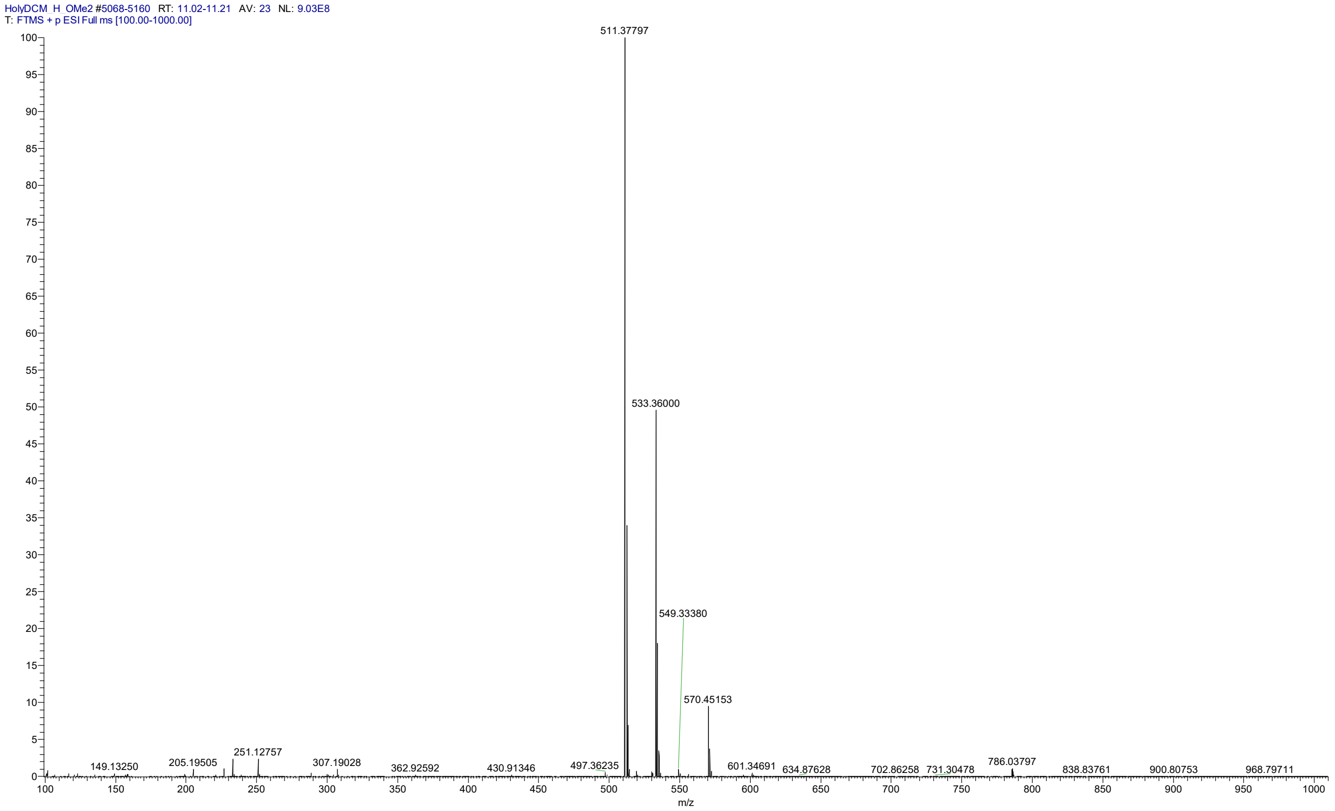

Figure S25. HRESIMS spectrum of compound **4b**.

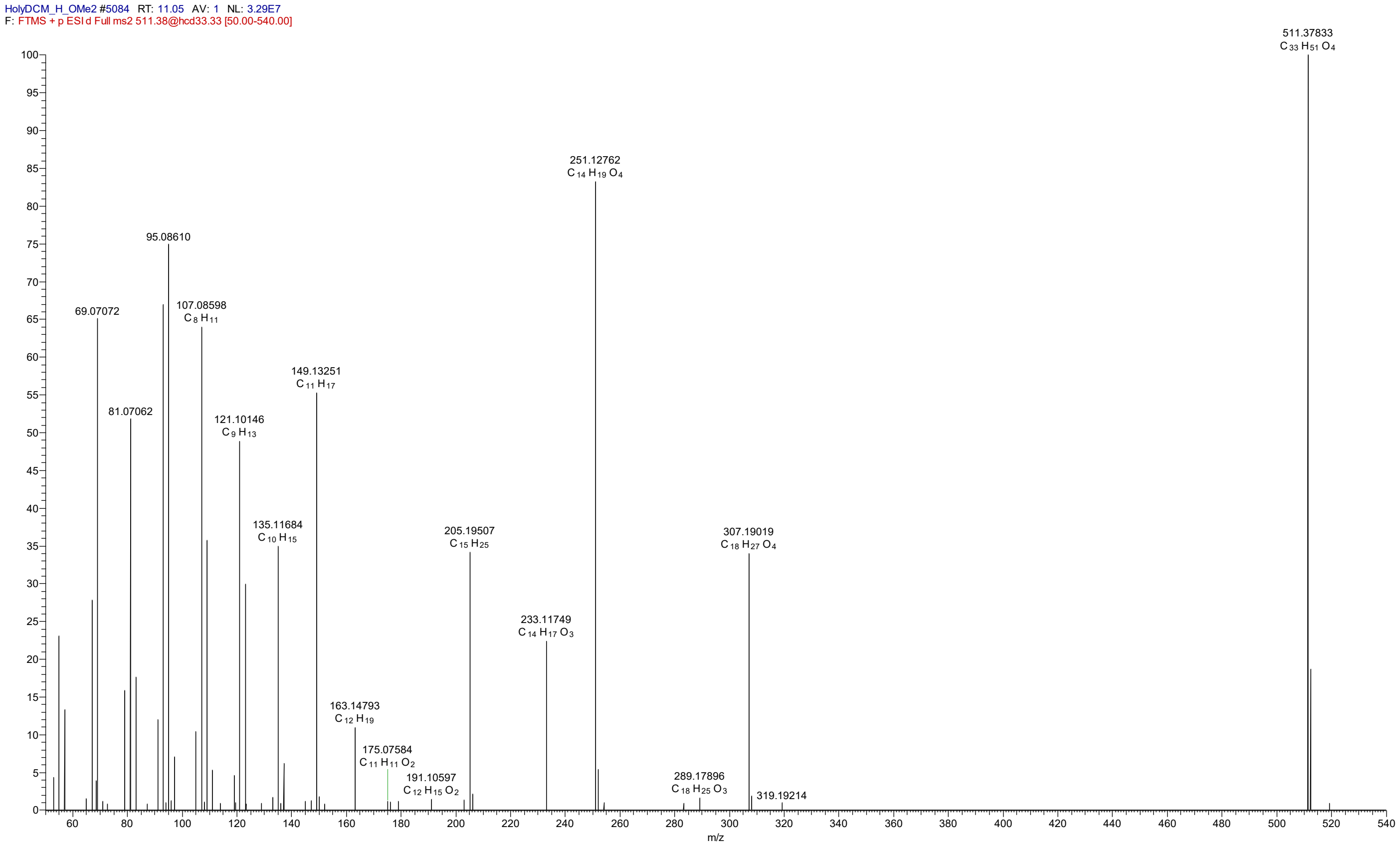

Figure S26. MS/MS spectrum of the protonated molecule $[M+H]^+$ of compound **4b**.

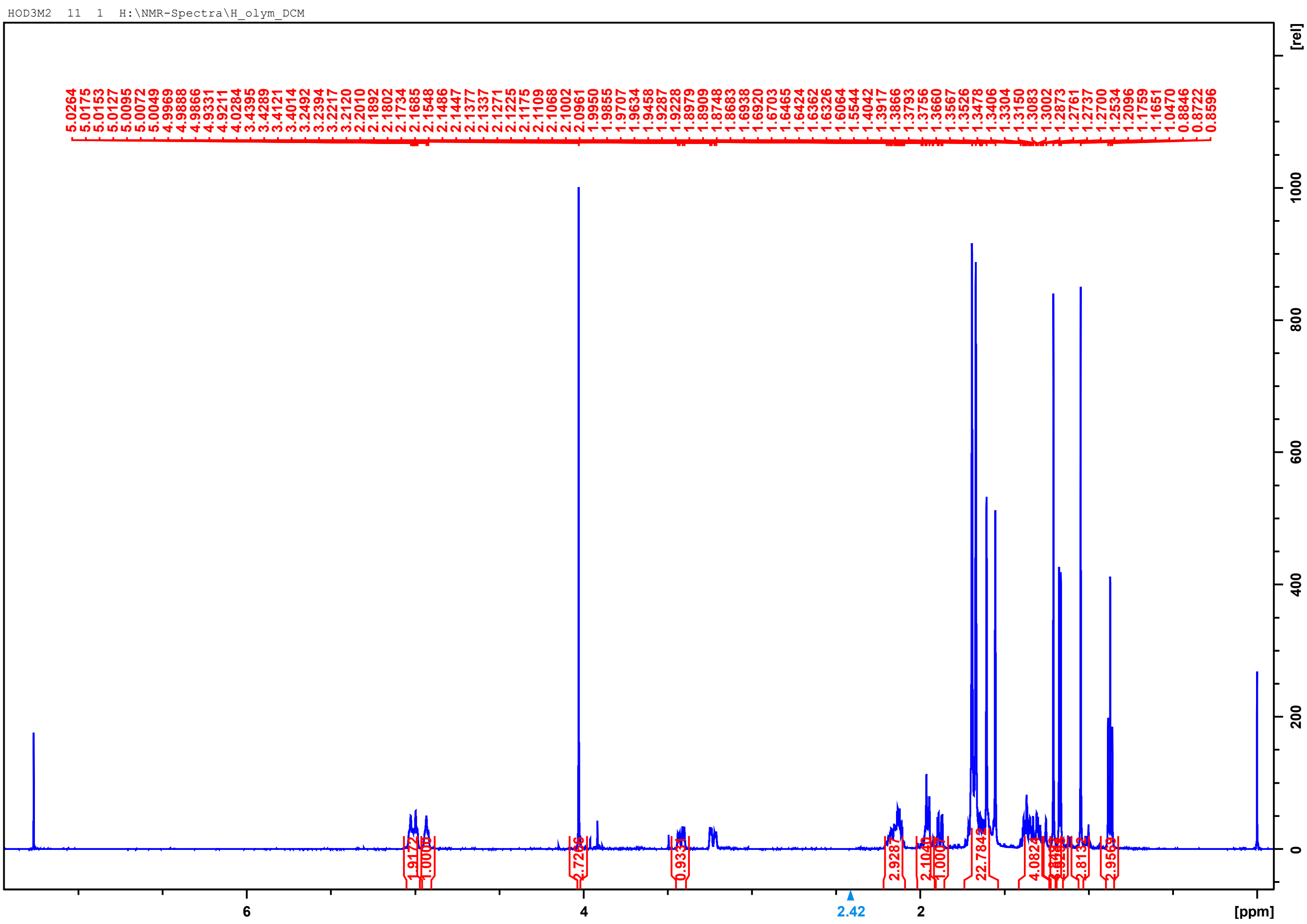

Figure S27. ^{1}H-NMR spectrum of compound **4b**.

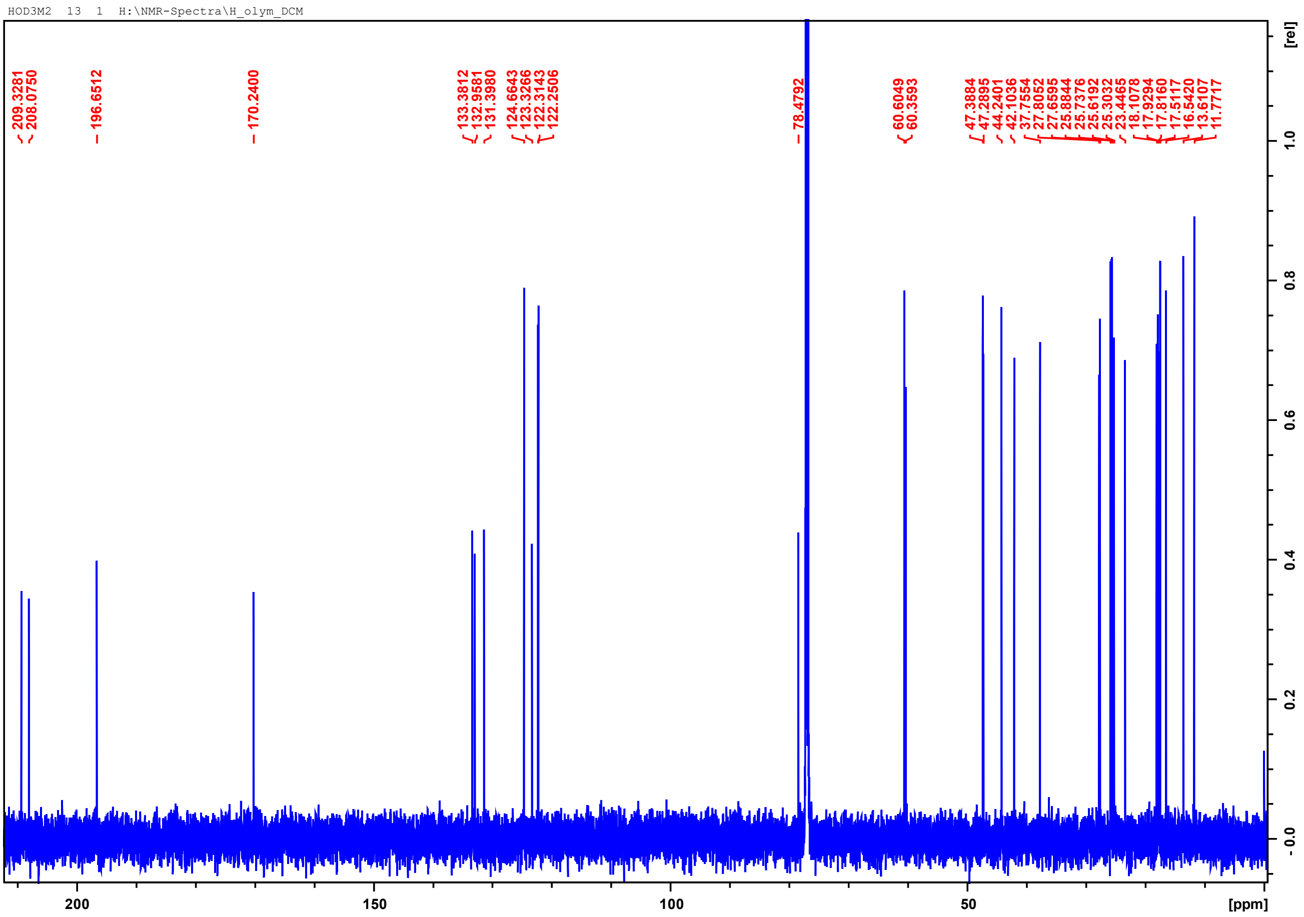

Figure S28. ^{13}C-NMR spectrum of compound **4b**.

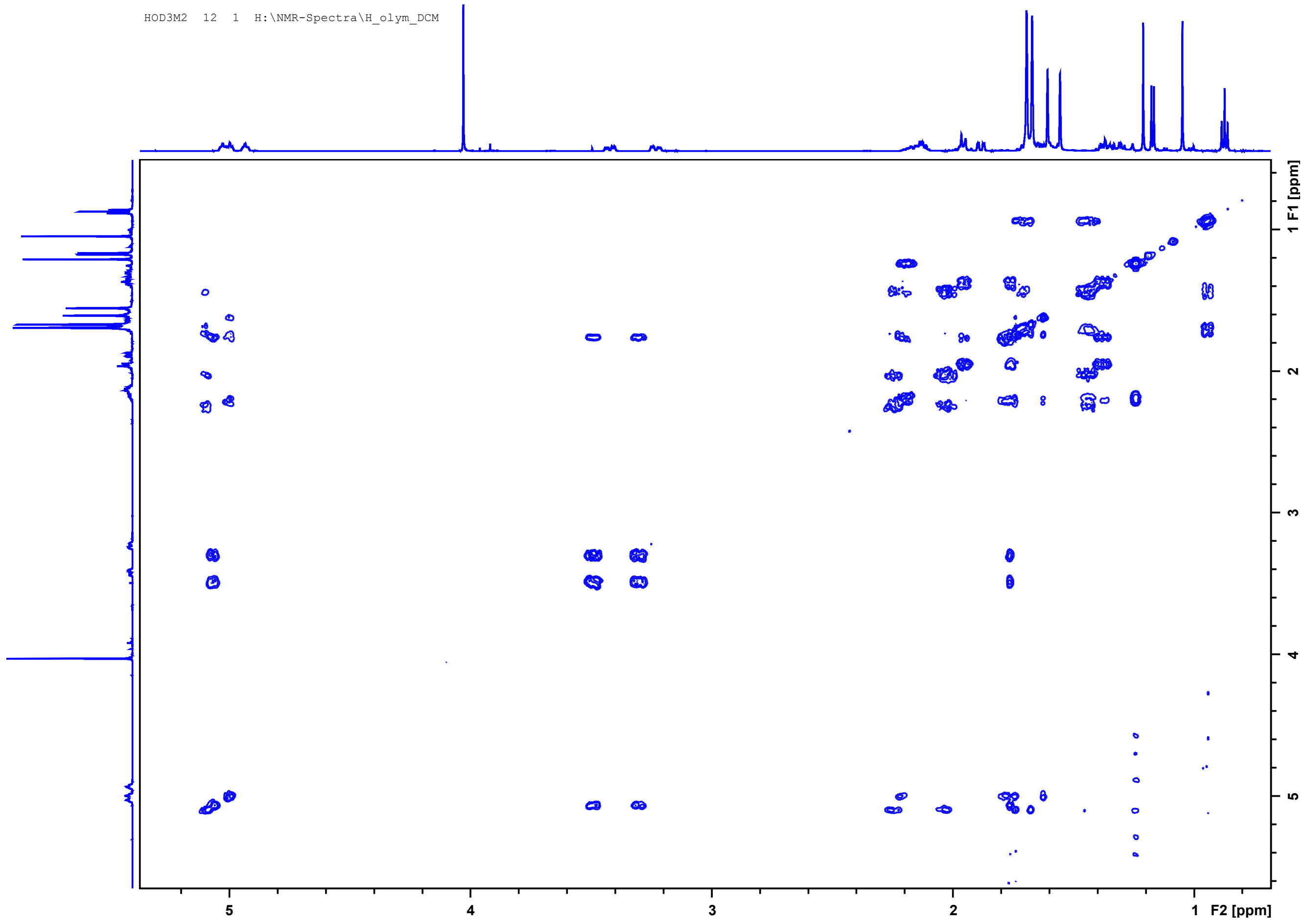

Figure S29. ^1H-^1H COSY experiment of compound **4a**.

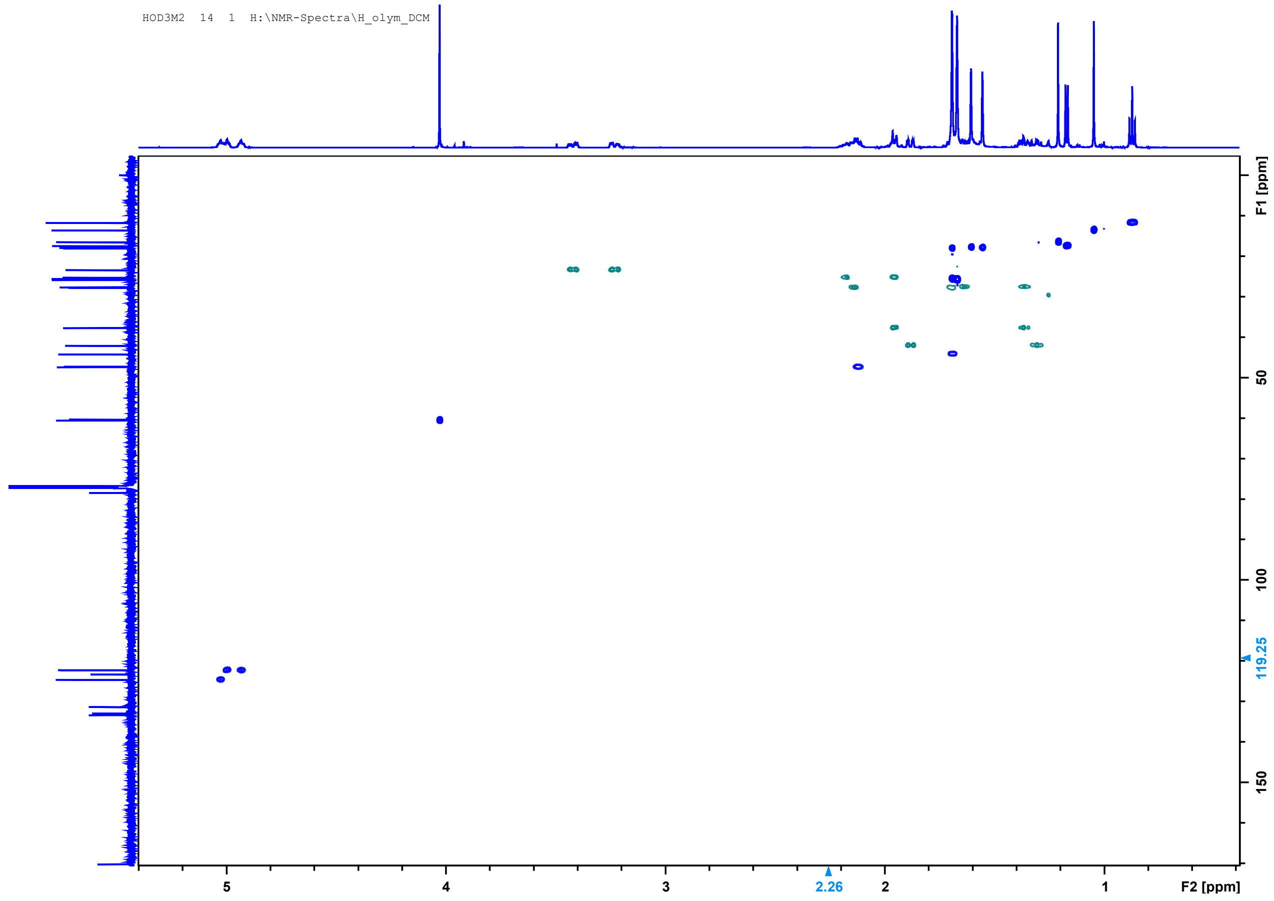

Figure S30. HSQC experiment of compound **4b**.

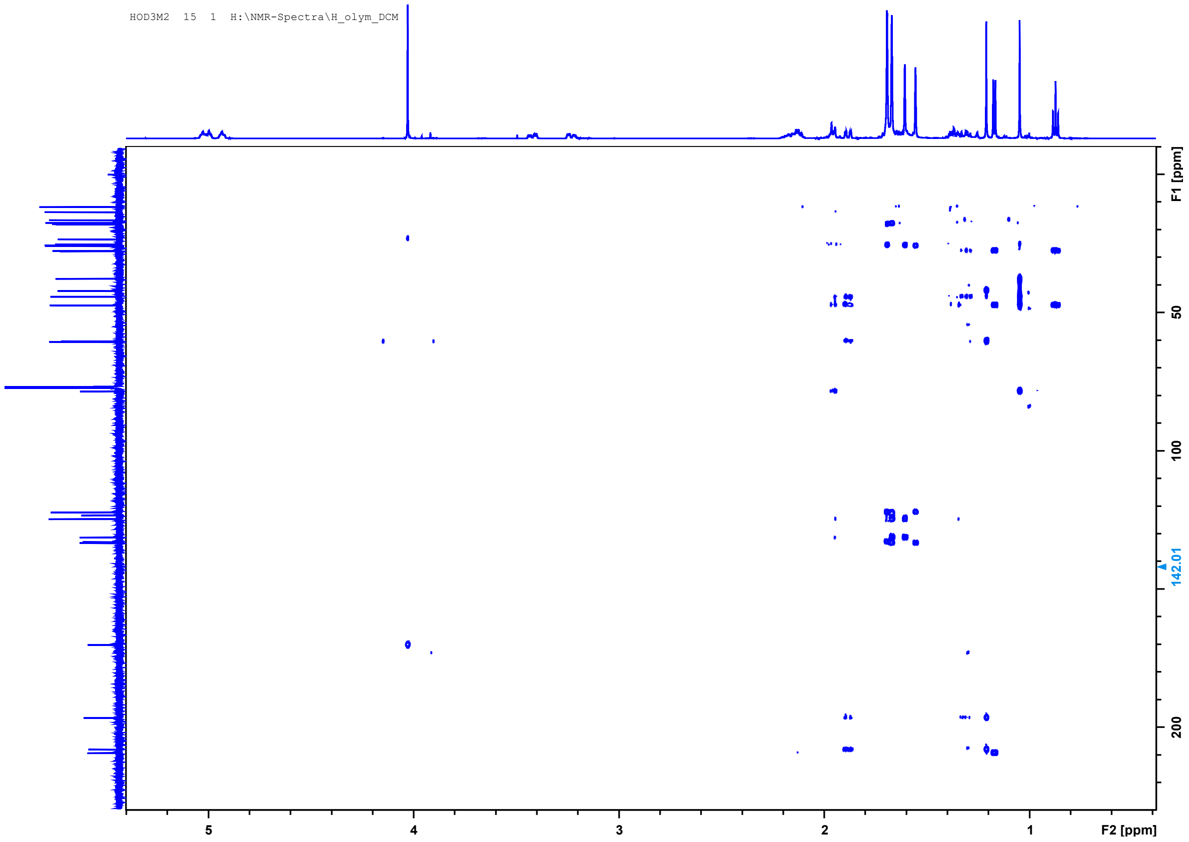

Figure S31. HMBC experiment of compound **4b**.

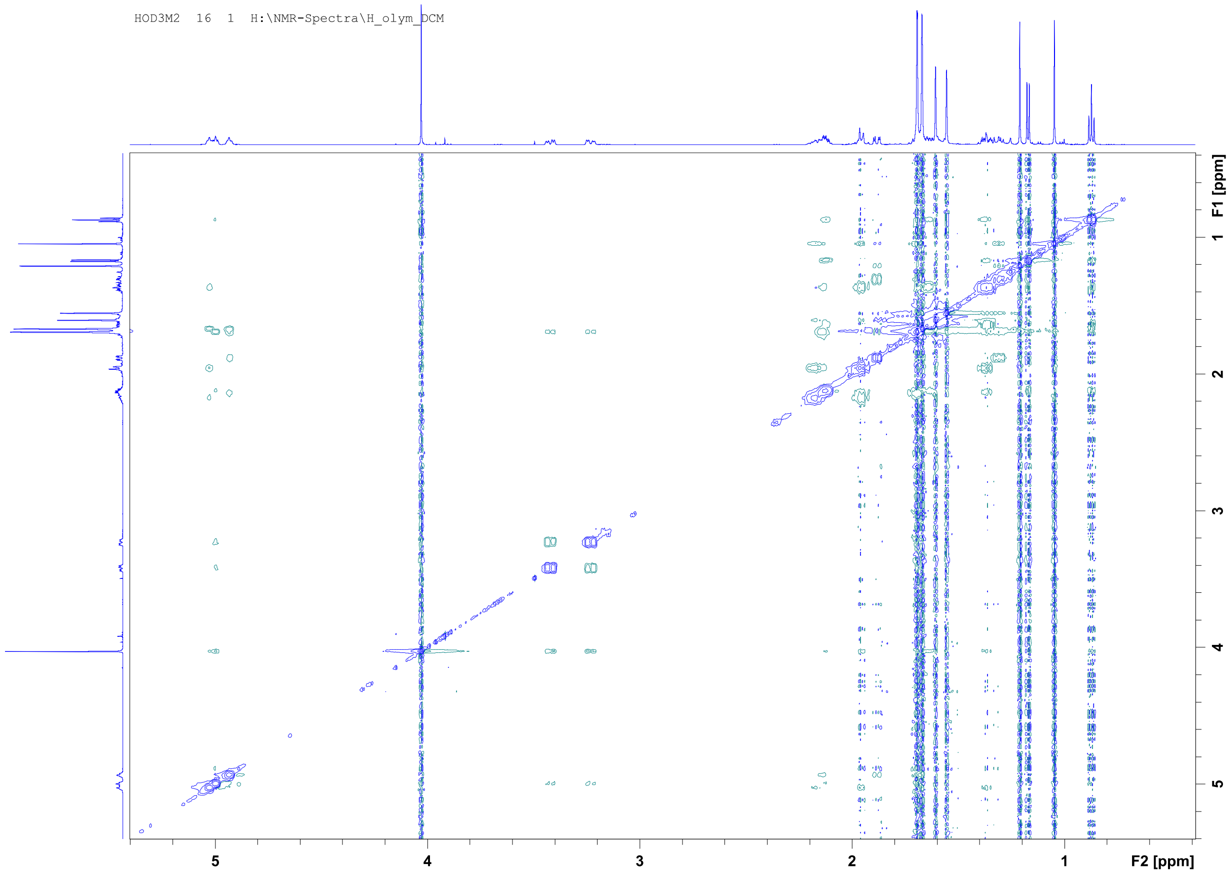

Figure S32. NOESY experiment of compound **4b**.

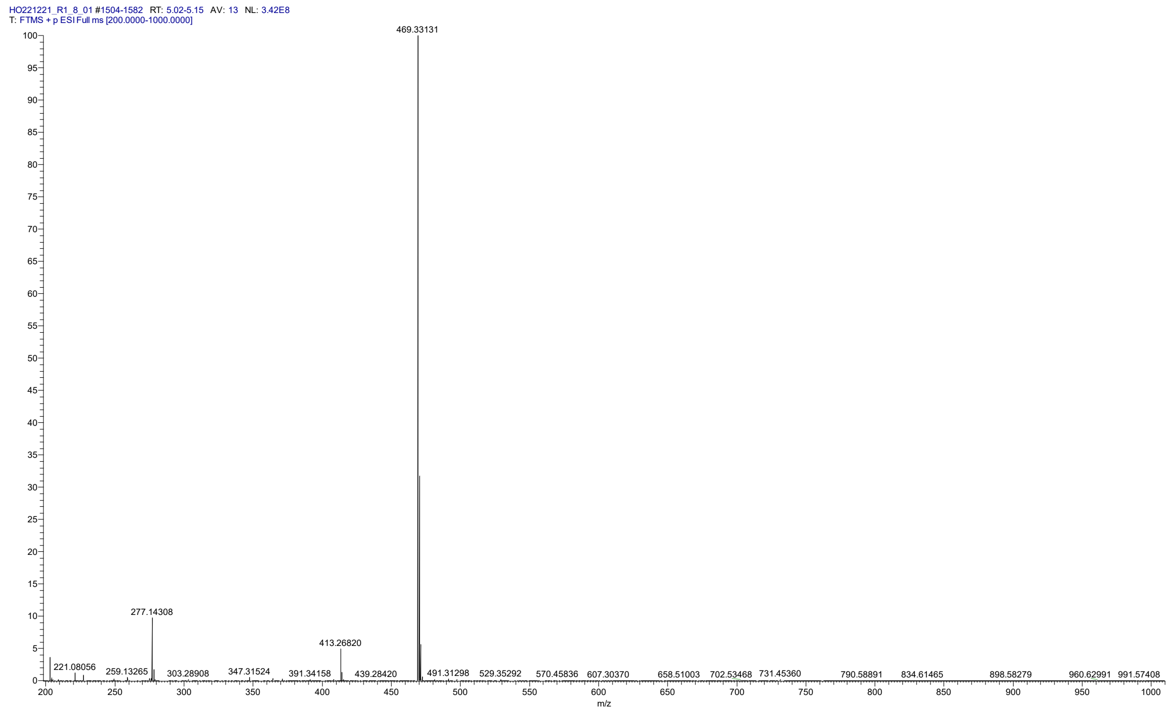

Figure S33. HRESIMS spectrum of compound **5**.

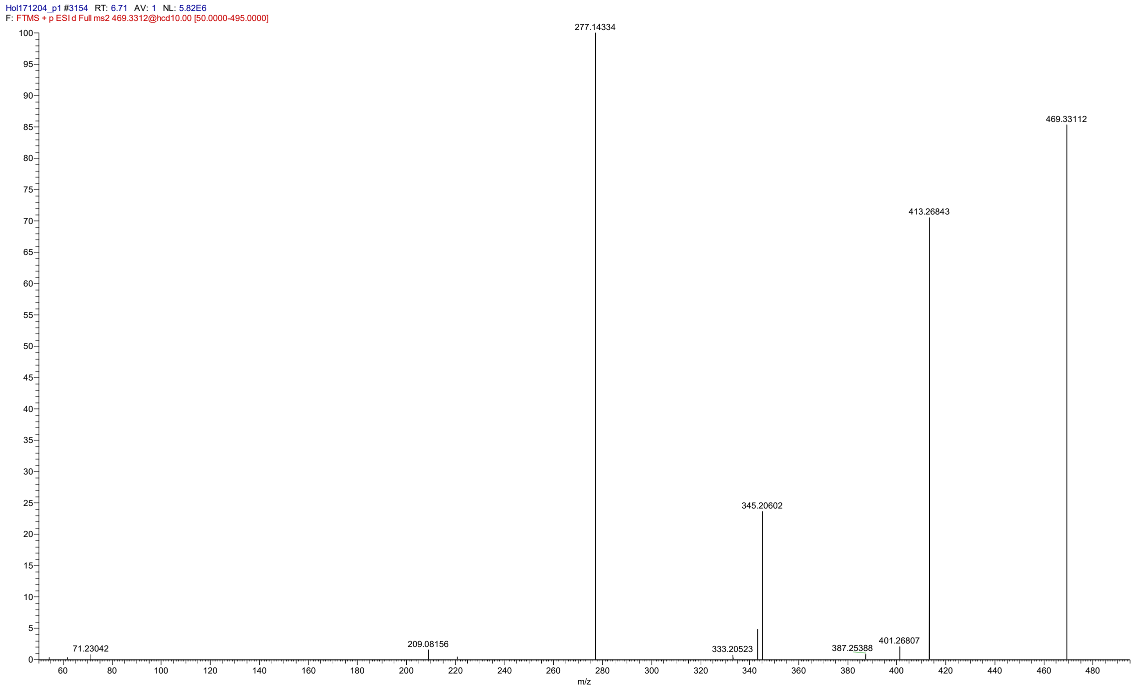

Figure S34. MS/MS spectrum of the protonated molecule $[M+H]^+$ of compound **5**.

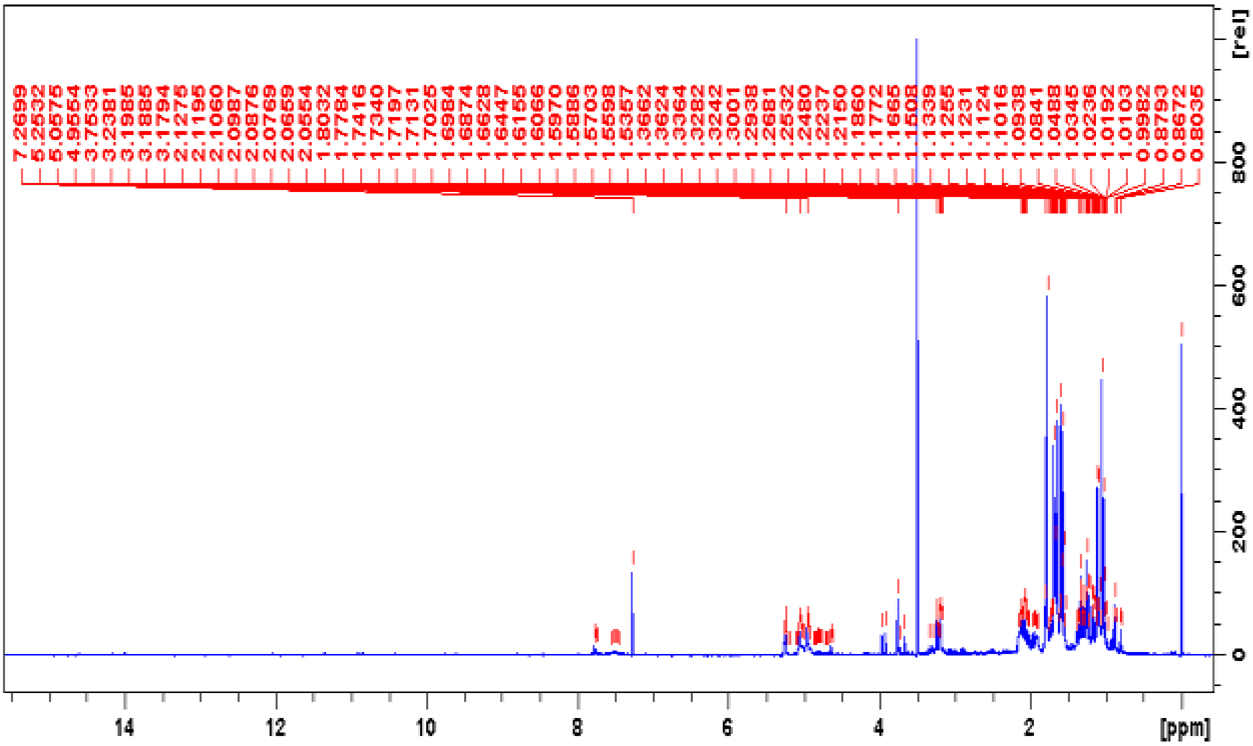

Figure S35. ^{1}H-NMR spectrum of compound **5**.

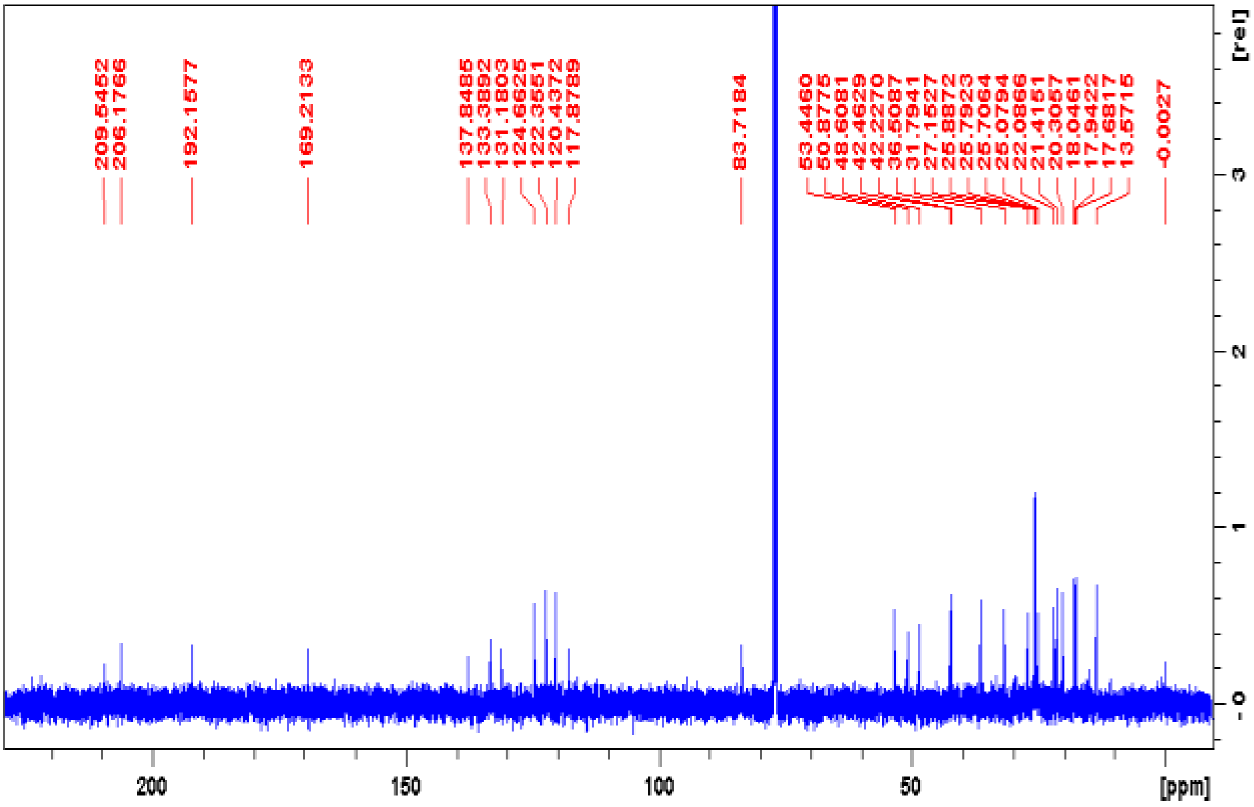

Figure S36.^{13}C-NMR spectrum of **5**.

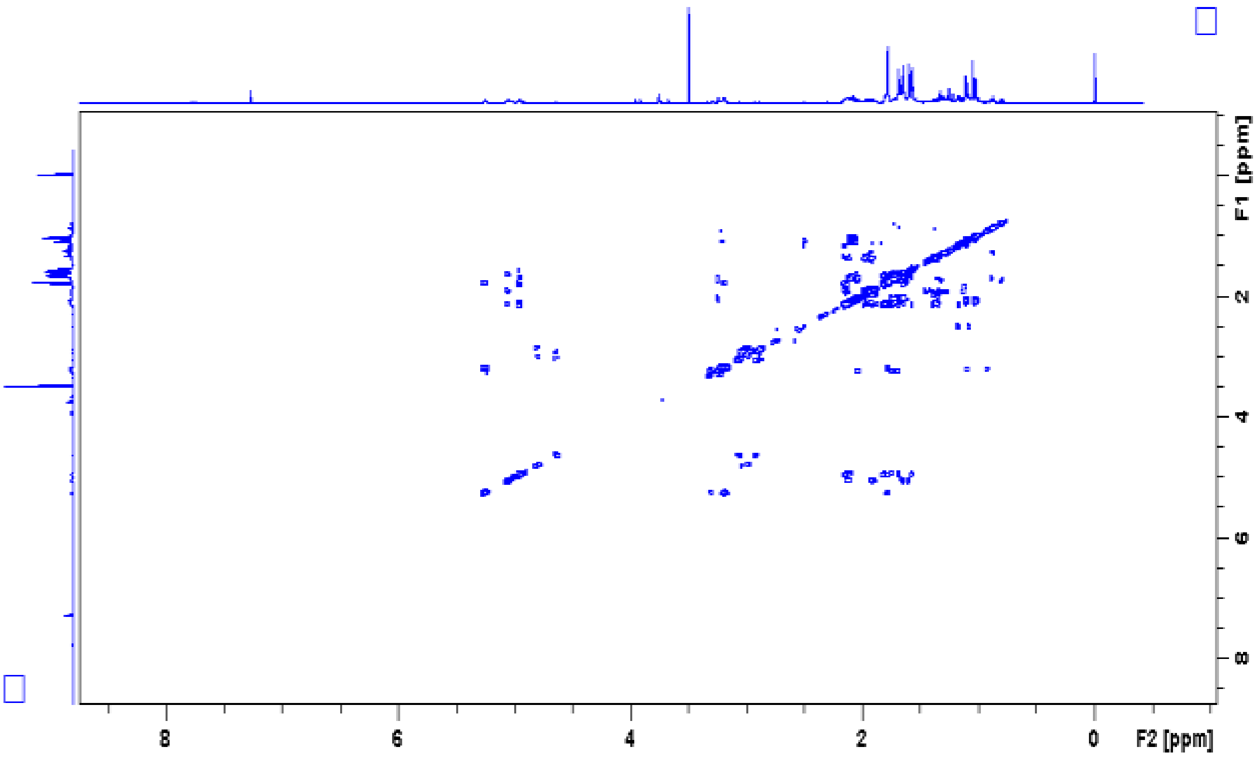

Figure S37. ^{1}H-^{1}H COSY experiment of compound **5**.

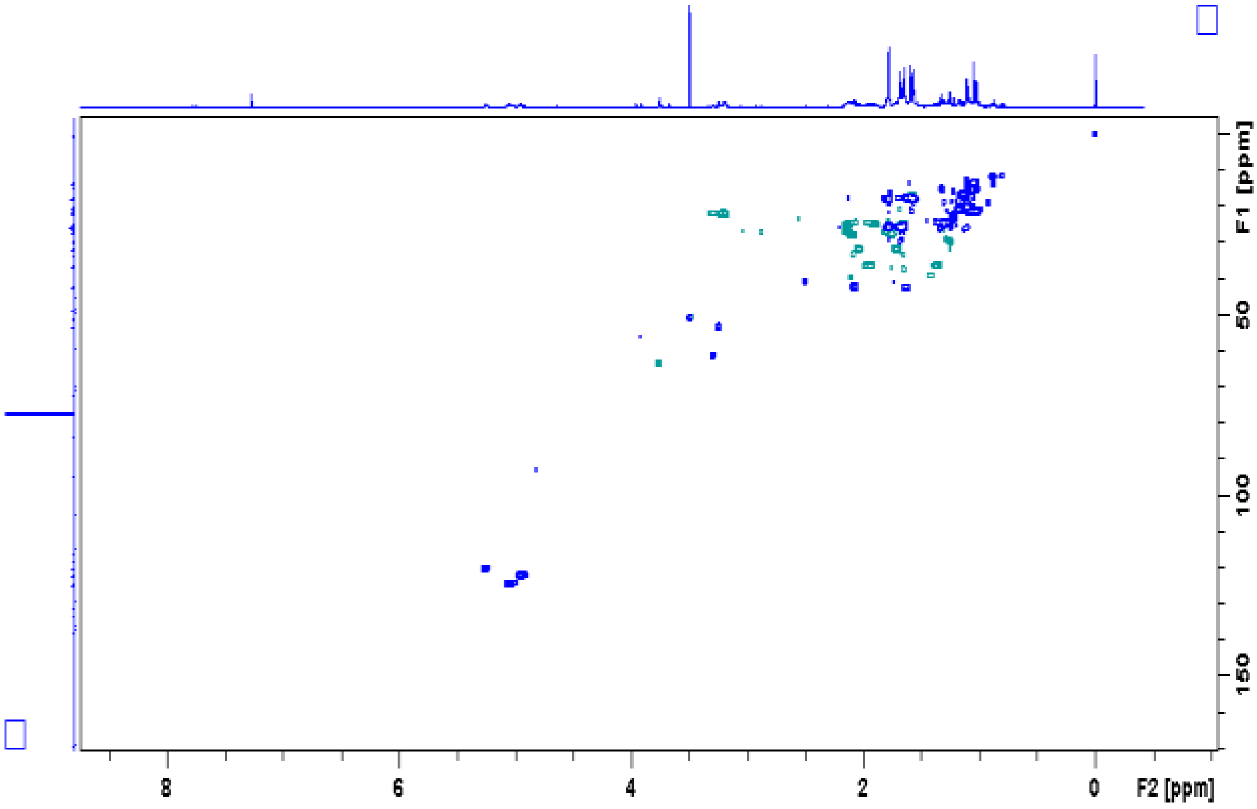

Figure S38. HSQC experiment of **5**.

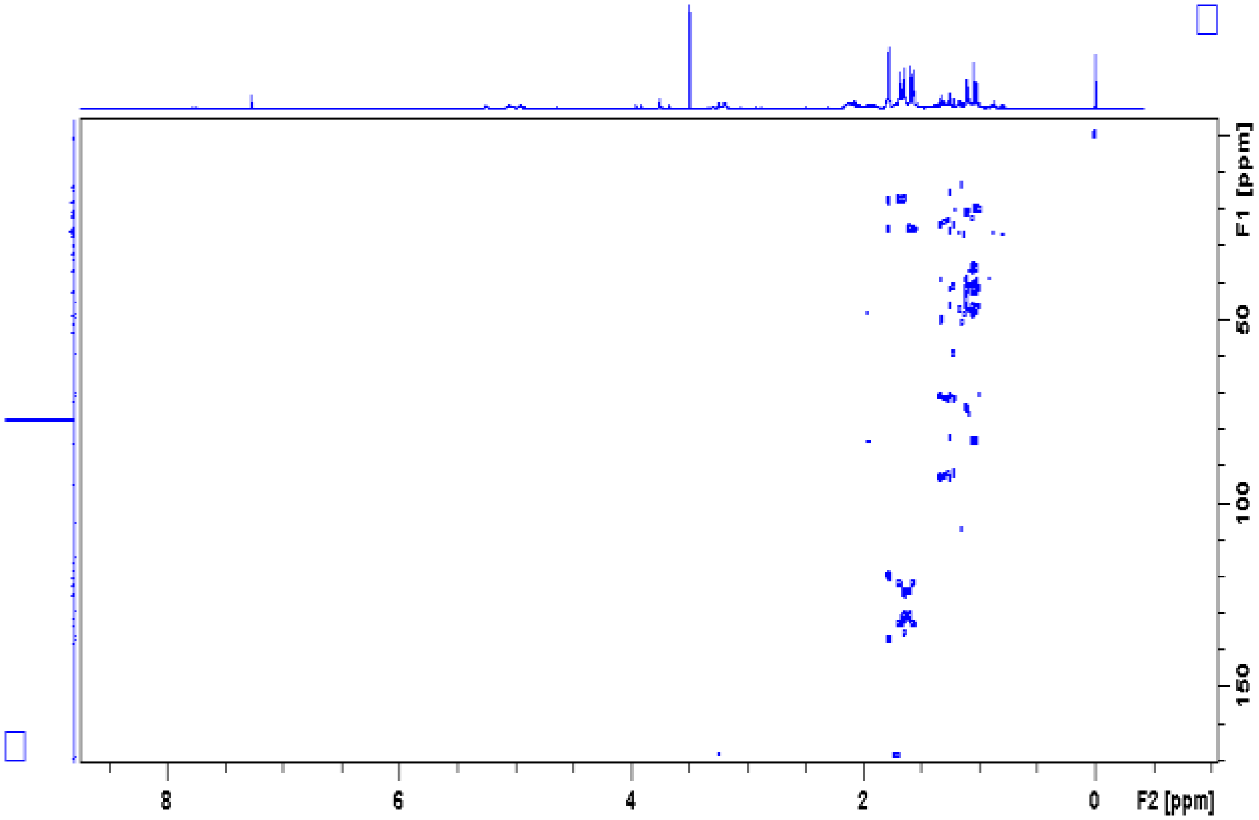

Figure S39. HMBC experiment of compound **5**.

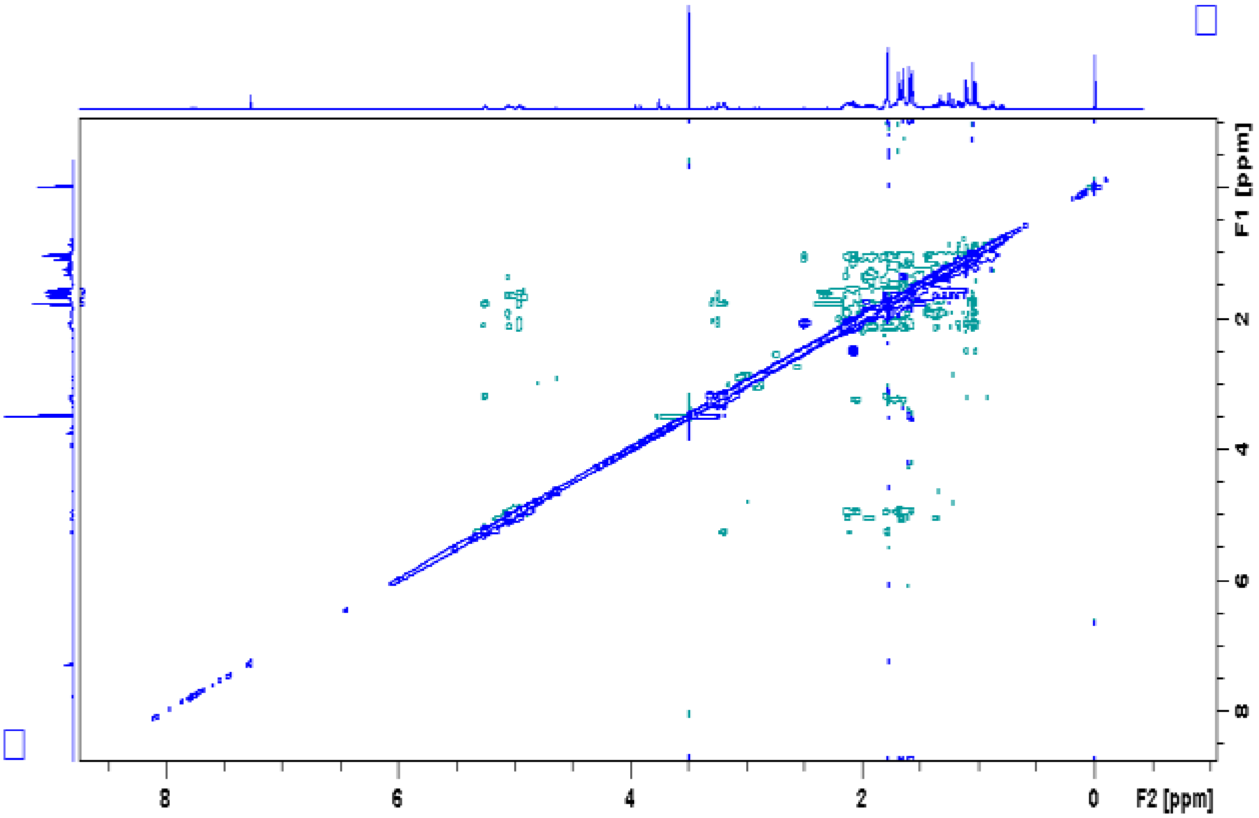

Figure S40. NOESY experiment of compound **5**.

Table S1. Absorbance at 550 nm of the MRSA biofilm died with Hucker crystal violet after exposure to the tested compounds.

Hyperpolyphyllirin/hyperibine J **(3)**					
Concentration [mg/L]	Absorbance				SD[1] %
0	0.672	0.786	0.802	0.671	11
0.125	0.347	0.336			1
0.25	0.376	0.403			3
0.5	0.276	0.394			13
1	0.249	0.184			7
2	0.444	0.353			10
4	0.110	0.099			1

Olympiforin A (**4**)					
Concentration [mg/L]	Absorbance				SD %
0	0.541	0.563	0.537	0.555	2
0.78	0.324	0.229	0.230		11
1.56	0.248	0.194	0.193		6
3.13	0.264	0,195	0,139		12
6.25	0.184	0.121	0.092		9
12.5	0.152	0.150	0.072		8

Olympiforin B (**5**)					
Concentration [mg/L]	Absorbance				SD %
0	2.066	2.116	2.050	2.218	4
0.063	0.978	1.044			2
0.125	0.926	0.790			5
0.25	0.592	0.502			3
0.5	0.534	0.342			7
1	0.406	0.332			2

[1] Standard deviation.

Table S2. One-way ANOVA of the metabolic activity of the tested strains after exposure to hyperpolyphyllirin/hyperibine J (**3**), olympiforin A (**4**) and olympiforin B (**5**). Comparison between the treated groups and untreated control.

Hyperpolyphyllirin/hyperibine J (3)				
**Bacteria**	**Dunnett's multiple comparisons test**	**Significance**		**Adjusted P Value**
S. aureus	Control vs. 100[1]	Yes	****	< 0.0001
	Control vs. 50	Yes	****	< 0.0001
	Control vs. 25	Yes	****	< 0.0001
	Control vs. 12.5	Yes	****	< 0.0001
	Control vs. 6.25	Yes	****	< 0.0001
	Control vs. 3.13	Yes	****	< 0.0001
	Control vs. 1.56	Yes	****	< 0.0001
	Control vs. 0.78	No	ns	0.9977
MRSA	Control vs. 100	Yes	****	< 0.0001
	Control vs. 50	Yes	****	< 0.0001
	Control vs. 25	Yes	****	< 0.0001
	Control vs. 12.5	Yes	****	< 0.0001
	Control vs. 6.25	Yes	****	< 0.0001
	Control vs. 3.13	Yes	****	< 0.0001
	Control vs. 1.56	Yes	****	< 0.0001
	Control vs. 0.78	Yes	**	0.0050
E. faecalis	Control vs. 100	Yes	****	< 0.0001
	Control vs. 50	Yes	****	< 0.0001
	Control vs. 25	Yes	****	< 0.0001
	Control vs. 12.5	Yes	****	< 0.0001
	Control vs. 6.25	No	ns	0.8631
	Control vs. 3.13	No	ns	0.9996
	Control vs. 1.56	No	ns	0.9394
	Control vs. 0.78	No	ns	> 0.9999
S. pyogenes	Control vs. 100	Yes	****	< 0.0001
	Control vs. 50	Yes	****	< 0.0001
	Control vs. 25	Yes	****	< 0.0001
	Control vs. 12.5	Yes	****	< 0.0001
	Control vs. 6.25	Yes	****	< 0.0001
	Control vs. 3.13	Yes	****	< 0.0001
	Control vs. 1.56	Yes	***	0.0002
	Control vs. 0.78	No	ns	0.7709

Olympiforin A (4)				
**Bacteria**	**Dunnett's multiple comparisons test**	**Significance**		**Adjusted P Value**
S. aureus	Control vs. 100	Yes	****	< 0.0001
	Control vs. 50	Yes	****	< 0.0001
	Control vs. 25	Yes	****	< 0.0001
	Control vs. 12.5	Yes	****	< 0.0001
	Control vs. 6.25	Yes	****	< 0.0001
	Control vs. 3.13	Yes	****	< 0.0001
	Control vs. 1.56	Yes	****	< 0.0001
	Control vs. 0.78	Yes	****	< 0.0001
	Control vs. 0.39	Yes	****	< 0.0001
	Control vs. 0.20	Yes	*	0.0353
MRSA	Control vs. 100	Yes	****	< 0.0001
	Control vs. 50	Yes	****	< 0.0001
	Control vs. 25	Yes	****	< 0.0001
	Control vs. 12.5	Yes	****	< 0.0001
	Control vs. 6.25	Yes	***	0.0003
	Control vs. 3.13	Yes	***	0.0003
	Control vs. 1.56	Yes	***	0.0003
	Control vs. 0.78	Yes	***	0.0007
	Control vs. 0.39	Yes	*	0.0167
	Control vs. 0.20	Yes	*	0.0107
E. faecalis	Control vs. 100	Yes	****	< 0.0001
	Control vs. 50	Yes	****	< 0.0001
	Control vs. 25	Yes	****	< 0.0001
	Control vs. 12.5	Yes	****	< 0.0001

	Control vs. 6.25	No	ns	0.9994
	Control vs. 3.13	No	ns	0.6106
	Control vs. 1.56	No	ns	0.8262
	Control vs. 0.78	No	ns	0.9996
	Control vs. 0.39	No	ns	0.9998
	Control vs. 0.20	No	ns	0.9558
S. pyogenes	Control vs. 100	Yes	****	< 0.0001
	Control vs. 50	Yes	****	< 0.0001
	Control vs. 25	Yes	****	< 0.0001
	Control vs. 12.5	Yes	****	< 0.0001
	Control vs. 6.25	Yes	****	< 0.0001
	Control vs. 3.13	Yes	****	< 0.0001
	Control vs. 1.56	Yes	****	< 0.0001
	Control vs. 0.78	Yes	****	< 0.0001
	Control vs. 0.39	Yes	****	< 0.0001
	Control vs. 0.20	Yes	****	< 0.0001

Olympiforin B (5)

Bacteria	Dunnett's multiple comparisons test	Significance		Adjusted P Value
S. aureus	Control vs. 100	Yes	****	< 0.0001
	Control vs. 50	Yes	****	< 0.0001
	Control vs. 25	Yes	****	< 0.0001
	Control vs. 12.5	Yes	****	< 0.0001
	Control vs. 6.25	Yes	****	< 0.0001
	Control vs. 3.13	Yes	****	< 0.0001
	Control vs. 1.56	Yes	****	< 0.0001
	Control vs. 0.78	Yes	****	< 0.0001
	Control vs. 0.39	Yes	****	< 0.0001
	Control vs. 0.20	Yes	***	0.0008
MRSA	Control vs. 100	Yes	****	< 0.0001
	Control vs. 50	Yes	****	< 0.0001
	Control vs. 25	Yes	****	< 0.0001
	Control vs. 12.5	Yes	****	< 0.0001
	Control vs. 6.25	Yes	****	< 0.0001
	Control vs. 3.13	Yes	****	< 0.0001
	Control vs. 1.56	Yes	****	< 0.0001
	Control vs. 0.78	Yes	****	< 0.0001
	Control vs. 0.39	Yes	****	< 0.0001
E. faecalis	Control vs. 100	Yes	****	< 0.0001
	Control vs. 50	Yes	****	< 0.0001
	Control vs. 25	Yes	****	< 0.0001
	Control vs. 12.5	Yes	****	< 0.0001
	Control vs. 6.25	Yes	***	0.0005
	Control vs. 3.13	Yes	*	0.0129
	Control vs. 1.56	No	ns	0.9997
	Control vs. 0.78	No	ns	0.6447
S. pyogenes	Control vs. 50	Yes	****	< 0.0001
	Control vs. 25	Yes	****	< 0.0001
	Control vs. 12.5	Yes	****	< 0.0001
	Control vs. 6.25	Yes	****	< 0.0001
	Control vs. 3.13	Yes	****	< 0.0001
	Control vs. 1.56	Yes	****	< 0.0001
	Control vs. 0.78	Yes	****	< 0.0001
	Control vs. 0.39	Yes	***	0.0008

[1] Concentration unit of all extracts is [mg/L]; ns – not significant.
